# Incidence, distribution, disease spectrum, and genetic deficits of congenital heart defects in China: implementation of prenatal ultrasound screening identified 18,171 affected fetuses from 2,452,249 pregnancies

**DOI:** 10.1186/s13578-023-01172-7

**Published:** 2023-12-19

**Authors:** Xinlin Chen, Sheng Zhao, Xiaoyan Dong, Juntao Liu, Yulin Guo, Weina Ju, Peiwen Chen, Yanduo Gao, Qian Feng, Xia Zhu, Hui Huang, Xiaojun Lu, Xiaohong Yang, Fan Yang, Chen Cheng, Xishun Luo, Longxian Cheng, Nanbert Zhong, On behalf of the Chinese Consortium for Prenatal Ultrasound Screening of Congenital Heart Defects

**Affiliations:** 1 Maternal and Child Health Hospital of Hubei Province, Wuhan, China; 2 New York State Institute for Basic Research in Developmental Disabilities, Staten Island, New York USA; 3 Department of Obstetrics and Gynecology, Peking Union Medical College Hospital, Chinese Academy of Medical Sciences & Peking Union Medical College, Beijing, China; 4 National Clinical Research Center for Obstetric & Gynecologic Diseases, Beijing, China

**Keywords:** Congenital heart disease, Ultrasound screening, Incidence, Spectrum, Genetic defects

## Abstract

**Background:**

Congenital heart defects (CHDs) are the most common birth defects. Assessment of the incidence, distribution, disease spectrum, and genetic deficits of fetal CHDs in China is urgently needed.

**Methods:**

A national echocardiography screening program for fetal CHDs was implemented in 92 prenatal screening–diagnostic centers in China.

**Findings:**

A total of 18,171 fetal CHD cases were identified from 2,452,249 pregnancies, resulting in 7·4/1,000 as the national incidence rate of fetal CHD. The incidences of fetal CHD in the six geographical regions, the southern, central, eastern, southwestern, northern, and northwestern, were 7·647 (CI: 7·383–7·915), 7·839 (CI: 7·680–8·000), 7·647 (CI: 7·383–7·915), 7·562 (CI: 7·225–7·907), 5·618 (CI: 5·337–5·906), and 4·716 (CI: 4·341–5·108), respectively, per 1,000 pregnancies. Overall, ventricular septal defect was the most common fetal CHD, accounting for 17.04% of screened pregnancies nationwide, and tetralogy of Fallot, the most common anomaly in the major defect of fetal CHD, was the second most common, accounting for 9.72%. A total of 76.24% cases of fetal CHD were found to be an isolated intracardiac single defect. The remaining 23.76% of cases of fetal CHD had multiple heart defects. Among all extracardiac malformations, the central nervous system (CNS) was the most common tissue with extracardiac anomalies associated with CHD, accounting for 22.89% of fetal CHD cases. Chromosomal karyotyping identified trisomy 18 as the most common chromosomal abnormality in fetal CHD. We also documented that CHD-containing syndromes could be identified with a comprehensive approach integrating prenatal ultrasound, MRI, pathological autopsy, and cytogenetics and molecular genetics.

**Conclusion:**

Implementation of prenatal echocardiography as a practically feasible platform to screen fetal CHD will reduce the financial and emotional burden of CHD, which may facilitate intrauterine and neonatal intervention of CHD.

**Supplementary Information:**

The online version contains supplementary material available at 10.1186/s13578-023-01172-7.

## Background

Congenital heart defects (CHDs), which result from incomplete or abnormal development of the fetal heart during the early stage of pregnancy, are the most common type of birth defect (https://www.cdc.gov/ncbddd/birthdefects/index.html), affecting 8 to 12 per 1,000 liveborn infants and accounting for nearly one-third of all major congenital anomalies worldwide [1, 2]. In the United States, about 40,000 (1%) births per year are affected with CHD (https://www.cdc.gov/ncbddd/heartdefects/data.html). Several studies of the regional incidence of CHD in China have shown that the incidence of CHDs was similar to that in the global report, [2–5] except that the incidence could be as high as 38.1 per 1,000 live births in the ethnic minority of “Uygur” at Xinjiang Uygur Autonomous Region (AR), which is geographically adjacent to Russia [6]. CHDs may result from genetic deficits; however, unexplained CHDs could occur secondarily to noncoding genetic, epigenetic, and environmental factors, among others [7–9]. Such environmental factors include abnormal development and dysfunction of the placenta in pathological pregnancies of intrauterine growth retardation, preterm birth, stillbirth, and preeclampsia [10–14]. Application of transabdominal echocardiography ultrasonographic screening to evaluate for CHDs in clinical practice has increased the detection rate of CHDs [15, 16]. Because of its ability to detect CHDs non-invasively in the early stage of pregnancy, prenatal ultrasound screening has been implemented, along with prenatal hemodynamic screening, as a routine clinical procedure in prenatal healthcare management worldwide [17].

Prenatal screening for CHDs, along with birth defects, has been implemented in China, along with the implementation of a surveillance system for public healthcare and prenatal healthcare [18]. Thousands of prenatal ultrasound specialists have been trained in the nationally credentialed training centers and have been certified for qualification to conduct prenatal ultrasound screening, which ensures that a standardized procedure is applied in the multicenter practices. However, detailed information about the true incidence and disease spectrum of CHDs throughout China is lacking. Therefore, most reports of the incidence/ prevalence and disease spectrum of CHDs in China identified from newborns before 2015 ignored fetuses with CHD that were aborted. In this first Chinese national study of the incidence and disease spectrum of CHDs, 18,171 fetal CHDs were identified and documented via fetal echocardiography through prenatal ultrasound screening for CHDs from among 2,452,249 pregnancies.

## Methods

### Research design and ethics issue

This project was a multi-institutional clinical research investigation by the Chinese Consortium for Prenatal Ultrasound Screening of Congenital Heart Defects, with fetal echocardiography to determine the national incidence, distribution, spectrum, and genetic defects of fetal CHD. The procedures and protocols implemented were reviewed and approved by the Ethics Committees of Maternal and Child Health Hospital of Hubei Province. The research studies were coordinated by the Department of Diagnostic Ultrasonography at the Maternal and Child Health Hospital of Hubei Province. Informed consent was obtained from each pregnant participant. Ultrasound data between the period of January 1, 2011 and December 31, 2013 were collected as part of clinical procedures for prenatal healthcare management.

### Implementation of prenatal screening of CHD with standardized procedures

As part of the ongoing national mandatory program to conduct nationwide surveillance for prevention of structural CHDs, the Ministry of Health (MOH)–credentialed National Prenatal Ultrasound Diagnostic Training Center (NPUDTC) initiated a training program in 2006 (Figure S1). A standardized operating procedure (SOP) for fetal echocardiography, following the guidelines of the International Society for Obstetrics and Gynecology (ISUOG) (http://www.ISUOG.org) and the American Institute of Ultrasound in Medicine (AIUM) Clinical Standards Committee, [19, 20] was provided to train prenatal ultrasound specialists. These trained specialists were capable of performing fetal ultrasound scan at the mid-trimester—optimally at between 18 and 22 weeks’ gestational age—with ultrasound skills for the four chambers, outflow tract including left ventricular outflow tract and right ventricular outflow tract, and three-vessel trachea views to capture and to recognize all types of CHD. Prenatal ultrasound specialists nationwide who graduated from and were certified by the NPUDTC for prenatal ultrasound screening of fetal CHD participated in this clinical research project among 93 prenatal screening-diagnostic centers (Table S1).

### Prenatal screening for CHD

Instruments used for prenatal ultrasound screening in this study included Siemens Acuson Sequoia 512 and S2000 (Siemens Medical Solutions Inc., Mountain View, CA, United States), Voluson 730 Expert and Voluson E8 (GE Healthcare, Kretz Ultrasound, Zipf, Austria), Philips IU 22 (Philips Medical Systems, Bothell, WA, United States), and Samsung UGEO WS80 (Samsung Medison Co., Ltd., Korea). In each ultrasound screening center, at least one advanced ultrasound system was employed. The final imaging was reviewed and confirmed by two ultrasound experts. The raw data were then transferred from the ultrasound unit directly into an electronic database to avoid generation of any errors via manual data entry. Random audits were performed to verify data reliability and accuracy during data transfer.

### Follow-up and confirmation

To verify the accuracy of prenatal ultrasound screening, positive cases determined by echocardiography were verified by postnatal follow-up with (1) clinical visits, (2) ultrasound and/or magnetic resonance imaging (MRI), (3) surgical observation, (4) pathological autopsy of aborted fetus, and (5) genetic studies.

### Data collection

Demographic information about pregnancies was collected from hospital administrative records. Software with multiple drop-down menus for the purpose of data collection from multi-centers was developed at the NPUDTC. Interpretations of fetal heart malformations followed the ISUOG and AIUM Clinical Standards Committee guidelines and were coded on the basis of the 10th revision of the International Statistical Classification of Diseases and Related Health Problems (ICD10) [17, 19–21]. CHD cases were grouped as CHDs with intra-cardiac malformation and CHDs with extra-cardiac malformation. The intra-cardiac malformations were further subgrouped into single abnormalities and multi-abnormalities. For multi-abnormalities, if more than one cardiac malformation was observed, the major anomaly was counted into the subgroup.

### Statistical analysis

All statistical analyses were performed using SPSS 13.0 software (SPSS Inc, Chicago, IL, USA) and Arcgis10.2. An unpaired t-test was used to compare incidence parameters among northern, central, eastern, western, and southern China. Categorical data were compared by using Pearson chi-square analysis. The relationship between incidence parameter and gross domestic product was assessed by simple linear regression analysis. *p* value of less than 0.05 was considered significant.

## Results/ findings

### Incidence of fetal CHD

**Fig. 1:**
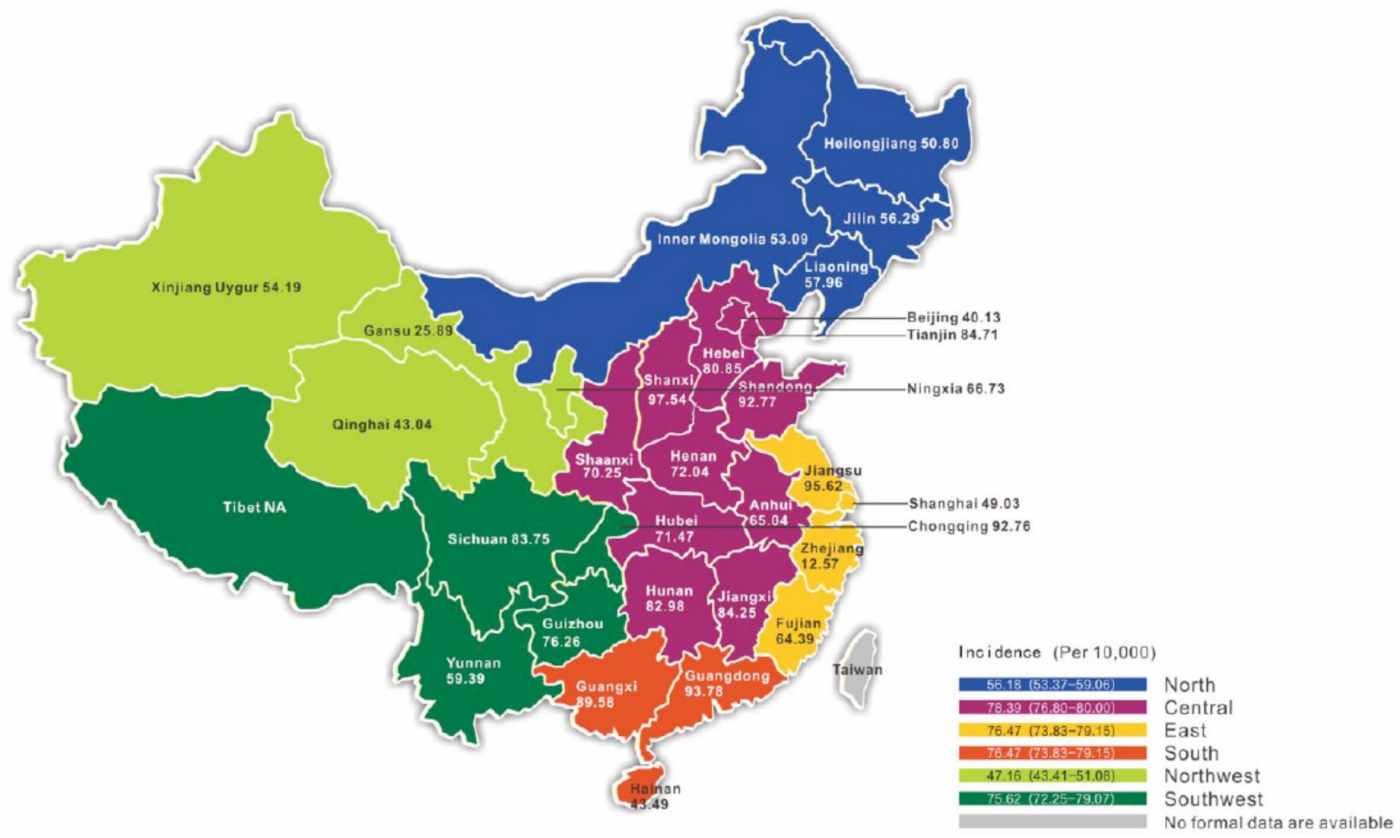
**Incidence of fetal CHD identified by prenatal echocardiography, and distribution across China**. Six regions, the north, central, east, south, northwest, and southwest, presented with dark blue, violet, yellow, red, light green, and dark green, respectively. Data from prenatal ultrasound screening for fetal CHD cases that were collected from Tibet were not available during quality control. No prenatal ultrasound screening data were collected from Taiwan

A total of 18,171 fetuses affected with CHDs were identified from 2,452,249 pregnancies (Table S2) among 92 (67 tertiary, 22 secondary, and three primary) hospitals across 31 provinces/municipalities/ ARs (Table 1a). Collection of reliable data was not successful in the Tibet AR (labeled as Xizang in Fig. 1), due to incomplete prenatal screening. The overall nationwide incidence of fetal CHD, with the lack of the Tibetanese population, was 74·099/10,000 (18,171/ 2,452,249) pregnancies in China. The regional incidences of fetal CHD in the six geographical regions—the southern, central, eastern, southwestern, northern, and northwestern—were 76·47 (CI: 73·83–79·15), 78·39 (CI: 76·80–80·00), 76·47 (CI: 73·83–79·15), 75·62 (CI: 72·25–79·07), 56·18 (CI: 53·37–59·06), and 47·16 (CI: 43·41–51·08) per 10,000 pregnancies. The incidences of fetal CHD in the ocean coast provinces, including the regions in the south and east, and the province of Shandong but not the province of Fujian or Zhejiang, were relatively higher than those in inland areas (Fig. 1). In the non–ocean coast region, the incidence of CHD was below 9·0 per 1,000 pregnancies in all provinces except Shanxi, which has the highest rate of neuronal tube defects (NTDs) in China and in the world. [22].

**Table 1a:** National incidence of variant subtypes of congenital heart defects in China during 2011–2013 (CI 95%)

Disease	Geographical area							Hospital level	
	North	Central	East	South	Northwest	Southwest	National	Tertiary	Primary/Secondary
Major									
TOF	9·03	10	12·34	8·42	5·6	7·97	9·72	9·04	0·69
	(7·92–10·20)	(9·44–10·58)	(11·30–13·44)	(7·25–9·67)	(4·36–7·00)	(6·91–9·12)	(9·34–10·12)	(8·66–9·42)	(0·59–0·79)
AVSD	5·73	8·11	6·68	7·97	4·95	6·69	7·29	6·78	0·51
	(4·86–6·67)	(7·60–8·63)	(5·91–7·49)	(6·84–9·18)	(3·79–6·27)	(5·72–7·75)	(6·96–7·63)	(6·46–7·11)	(0·42–0·60)
DORV	3·78	5·71	4·65	6·12	3·82	4·01	5·09	4·85	0·24
	(3·08–4·56)	(5·28–6·15)	(4·02–5·33)	(5·14–7·19)	(2·80–4·99)	(3·26–4·83)	(4·81–5·38)	(4·58–5·13)	(0·18–0·30)
TGA	2·85	3·69	4·72	4·37	2·76	4·01	3·82	3·6	0·22
	(2·24–3·52)	(3·35–4·05)	(4·09–5·41)	(3·54–5·28)	(1·91–3·77)	(3·26–4·83)	(3·58–4·07)	(3·36–3·84)	(0·17–0·29)
SV	1·65	3·79	0·75	4·46	2·52	5·33	3·19	3·01	0·18
	(1·20–2·17)	(3·44–4·15)	(0·51–1·03)	(3·62–5·38)	(1·71–3·48)	(4·46–6·27)	(2·97–3·42)	(2·8–3·23)	(0·13–0·24)
HLHS	2·4	2·66	3·54	2·66	2·03	3·41	2·83	2·7	0·13
	(1·85–3·02)	(2·38–2·97)	(2·99–4·14)	(2·02–3·38)	(1·31–2·90)	(2·72–4·17)	(2·62–3·04)	(2·5–2·91)	(0·09–0·17)
PTA	3·37	2·57	2·15	2·57	2·44	3·89	2·71	2·55	0·16
	(2·71–4·10)	(2·29–2·87)	(1·72–2·61)	(1·94–3·28)	(1·64–3·39)	(3·15–4·70)	(2·51–2·92)	(2·35–2·75)	(0·12–0·22)
HRHS	1·12	2·24	2·15	2·66	1·14	2·4	2·1	2	0·1
	(0·76–1·56)	(1·98–2·52)	(1·72–2·61)	(2·02–3·38)	(0·62–1·81)	(1·84–3·05)	(1·92–2·29)	(1·83–2·18)	(0·06–0·14)
HS	1·2	2·03	2·19	2·07	0·81	1·0	1·81	1·72	0·09
	(0·82–1·65)	(1·78–2·30)	(1·77–2·67)	(1·52–2·71)	(0·39–1·39)	(0·65–1·43)	(1·64–1·98)	(1·56–1·88)	(0·06–0·13)
PA	0·07	0·7	0·72	0·68	0·24	0·24	0·56	0·55	0·01
	(0·01–0·22)	(0·55–0·86)	(0·49–1·01)	(0·38–1·06)	(0·05–0·60)	(0·09–0·47)	(0·47–0·66)	(0·46–0·65)	(0·00–0·03)
Other major*	0·41	0·62	1·01	0·95	0·57	1·2	0·75	0·68	0·07
	(0·21–0·69)	(0·49–0·77)	(0·73–1·34)	(0·58–1·39)	(0·23–1·07)	(0·81–1·67)	(0·65–0·86)	(0·58–0·78)	(0·04–0·11)
Minor									
VSD	11·57	19·31	20·59	14·31	8·85	19·44	17·72	17·04	0·68
	(10·32–12·9)	(18·52–20·11)	(19·23–21·99)	(12·78–15·93)	(7·27–10·59)	(17·75–21·20)	(17·20–18·25)	(16·53–17·56)	(0·58–0·78)
PLSVC	3·26	4·22	3·83	9·05	2·11	2·77	4·23	3·84	0·4
	(2·61–3·98)	(3·86–4·60)	(3·26–4·45)	(7·84–10·34)	(1·38–3·00)	(2·15–3·46)	(3·98–4·49)	(3·6–4·09)	(0·32–0·48)
COA/IAA	1·42	2·75	2·53	4·64	2·52	2·64	2·72	2·6	0·11
	(1·01–1·91)	(2·46–3·06)	(2·07–3·04)	(3·78–5·58)	(1·71–3·48)	(2·05–3·32)	(2·51–2·93)	(2·40–2·81)	(0·08–0·16)
AS	2·4	1·5	2·43	2·25	1·06	2·48	1·9	1·81	0·09
	(1·85–3·02)	(1·28–1·73)	(1·98–2·93)	(1·67–2·92)	(0·56–1·71)	(1·91–3·14)	(1·73–2·08)	(1·65–1·99)	(0·05–0·13)
PS	1·2	1·86	2·22	1·8	1·38	1·76	1·81	1·67	0·14
	(0·82–1·65)	(1·62–2·12)	(1·79–2·69)	(1·29–2·40)	(0·80–2·12)	(1·28–2·32)	(1·65–1·98)	(1·51–1·83)	(0·10–0·19)
CVR	0·6	2·25	0·63	1·76	0·41	1·32	1·57	1·43	0·14
	(0·34–0·93)	(1·99–2·53)	(0·41–0·89)	(1·25–2·35)	(0·13–0·84)	(0·91–1·81)	(1·41–1·73)	(1·28–1·58)	(0·10–0·19)
Rhabdomyoma	1·09	1·17	1·04	1·62	1·06	1·56	1·21	1·13	0·09
	(0·73–1·52)	(0·98–1·37)	(0·75–1·37)	(1·14–2·19)	(0·56–1·71)	(1·11–2·09)	(1·08–1·35)	(1·00–1·26)	(0·05–0·13)
RAA	1·84	0·53	0·84	1·94	1·38	1·52	1·0	0·94	0·06
	(1·36–2·39)	(0·40–0·67)	(0·59–1·15)	(1·40–2·56)	(0·80–2·12)	(1·08–2·05)	(0·87–1·12)	(0·82–1·06)	(0·03–0·09)
TVS	0·56	1·16	0·48	0·45	0·81	0·56	0·84	0·8	0·04
	(0·31–0·88)	(0·97–1·36)	(0·29–0·72)	(0·21–0·77)	(0·39–1·39)	(0·31–0·89)	(0·73–0·95)	(0·69–0·91)	(0·02–0·07)
EA	0·37	0·61	0·31	0·41	0·49	0·64	0·51	0·5	0·01
	(0·18–0·64)	(0·48–0·76)	(0·17–0·51)	(0·18–0·71)	(0·18–0·96)	(0·37–0·99)	(0·43–0·61)	(0·42–0·59)	(0·00–0·03)
Other minor^†^	0·26	0·92	0·65	0·63	0·24	0·76	0·73	0·7	0·03
	(0·10–0·49)	(0·75–1·10)	(0·43–0·92)	(0·34–1·00)	(0·05–0·60)	(0·46–1·14)	(0·62–0·84)	(0·60–0·81)	(0·01–0·05)

Other major*: Other major anomalies including total anomalous pulmonary venous drainage, double-outlet left ventricle, aortic atresia, APW, ectopia cordis

Other minor^†^: Other minor anomalies including tricuspid atresia, mitral valve stenosis, coronary artery fistula, conjoined twins with a heart, mitral atresia, cor triatriatum, ventricular aneurysm, endocardial fibroelastosis, premature closure of the ductus arteriosus, bicuspid aortic valve, cardiac diverticulum

APW = aortopulmonary window. AS = aortic stenosis. AVSD = atrioventricular septal defect. CI = confidence interval. COA/IAA = coarctation of the aorta/interrupted aortic arch. CVR = congenital vascular ring. DORV = double-outlet right ventricle. EA = Ebstein's anomaly. HLHS = hypoplastic left heart syndrome. HRHS = hypoplastic right heart syndrome. HS = heterotaxy syndrome. PA = pulmonary atresia. PLSVC = persistent left superior vena cava. PS = pulmonary stenosis. PTA = persistent truncus arteriosus. RAA = right aortic arch. SV = single ventricle. TGA = transposition of the great arteries. TOF = tetralogy of Fallot. TVS = tricuspid valve stenosis. VSD = ventricular septal defect

### Distribution of CHD

**Fig. 2:**
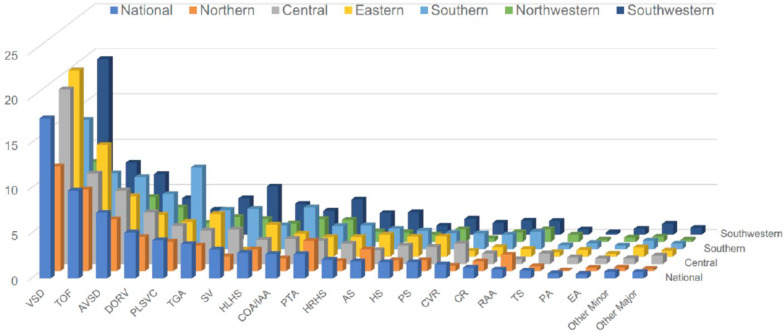
**Subtypes of fetal CHD and their proportionsf in six regions and nation-wide**. VSD was determined to be the most common subtype of fetal CHD, followed by TOF as the second most common subtype. AS, aortic stenosis. AVSD, atrioventricular septal defect. COA/IAA, coarctation of the aortic arch/interrupted aortic arch. CVR, congenital vascular ring. DORV, double-outlet rightventricle. EA, Ebstein's anomaly. HLHS, hypoplastic left heart syndrome. HRHS, hypoplastic right heart syndrome. HS, heterotaxy syndrome. PA, pulmonary atresia. PLSVC, persistent left superior vena cava. PS, pulmonary stenosis. PTA, persistent truncus arteriosus. RAA, right aortic arch. SV, single ventricle. TGA, transposition of the great arteries. TOF, tetralogy of Fallot. VSD, ventricular septal defect

Subtypes of CHD in the major and minor anomalies and their presence in tertiary hospitals vs. in primary and secondary hospitals were documented (Table 1a). Overall, ventricular septal defect (VSD) was the most common fetal CHD, accounting for 17·04% of pregnancies screened nationwide. Tetralogy of Fallot (TOF), the most common anomaly in the major defect of fetal CHD, was the second most common after VSD, accounting for 9·72% of pregnancies. Following VSD and TOF, the incidences of CHD were atrioventricular septal defect (AVSD) (7·29%), double-outlet right ventricle (DORV) (5·09%), persistent left superior vena cava (PLSVC) (4·23%), transposition of the great artery (TGA) (3·82%), single ventricle (SV) (3·19%), hypoplastic left heart syndrome (HLHS) (2·83%), coarctation of the aortic arch/interrupted aortic arch (COA/IAA) (2·72%), and persistent truncus arteriosus (PTA) (2·71%). These CHDs comprised the top-10 most common fetal CHDs (Fig. 2).

### Spectrum of all anomalies of CHD

The spectrum of CHD cases in China was determined to comprise 15 major and 21 minor anomalies (Table 1b). Among the 18,171 fetal CHD cases, 14,096 (76·24%) were isolated single defects within the heart, the top 5 most common of which were VSD (29·09%), TOF (14·43%), AVSD (10·24), PLSVC (7·36%), and TGA (4·51%). The remaining 4,075 (23·76%) cases had multiple heart defects, the top 5 most common of which were DORV (18·58%), SV (12·29%), COA/IAA (10·77%), TOF (8·59%), and AVSD (8·44%).

**Table 1b:** Spectrum and proportion of congenital heart defect subtypes

	CHD subtype	Single anomaly	Multi-anomalies	Subtotal	Subtype in major / minor anomaly	Subtype in all CHDs identified
Major	Subtotal	6,947	2,831	9,778	100.00%	53.81%
1	TOF	2,034	350	2,384	24·38%	13·12%
2	AVSD	1,444	344	1,788	18·29%	9·84%
3	DORV	491	757	1,248	12·76%	6·87%
4	TGA	636	301	937	9·58%	5·16%
5	SV	282	501	783	8·01%	4·31%
6	HLHS	587	106	693	7·09%	3·81%
7	PTA	415	250	665	6·80%	3·66%
8	HRHS	451	64	515	5·27%	2·83%
9	HS	443	0	443	4·53%	2·44%
10	PA	75	63	138	1·41%	0·76%
11	TAPVD	47	33	80	0·82%	0·44%
12	DOLV	6	26	32	0·33%	0·18%
13	AA	5	21	26	0·27%	0·14%
14	APW	9	14	23	0·24%	0·13%
15	EC	22	1	23	0·24%	0·13%
**Minor**	**Subtotal**	**7,149**	**1,244**	**8,393**	**100·00%**	**46·19%**
1	VSD	4,100	245	4,345	51·77%	23·91%
2	PLSVC	1,037	1	1,038	12·37%	5·71%
3	COA/IAA	227	439	666	7·94%	3·67%
4	AS	131	335	466	5·55%	2·56%
5	PS	422	22	444	5·29%	2·44%
6	CVR	378	6	384	4·58%	2·11%
7	Rhabdomyoma	283	14	297	3·54%	1·63%
8	RAA	231	13	244	2·91%	1·34%
9	TVS	140	65	205	2·44%	1·13%
10	EA	70	56	126	1·50%	0·69%
11	TA	23	17	40	0·48%	0·22%
12	MVS	25	8	33	0·39%	0·18%
13	CAF	15	8	23	0·27%	0·13%
14	CTOH	17	0	17	0·20%	0·09%
15	MA	9	6	15	0·18%	0·08%
16	CT	11	2	13	0·15%	0·07%
17	VA	9	1	10	0·12%	0·06%
18	EFE	6	5	11	0·13%	0·06%
19	PCDA	7	1	8	0·10%	0·04%
20	BAV	4	0	4	0·05%	0·02%
21	CD	4	0	4	0·05%	0·02%
	Total	14,096	4,075	18,171		
	Percentage	76·24%	23·76%			

AA = aortic atresia. APW = aortopulmonary window. AS = aortic stenosis. AVSD = atrioventricular septal defect. BAV = bicuspid aortic valve. CAF = coronary artery fistula. CD = cardiac diverticulum. CHD = congenital heart defect. COA/IAA = coarctation of the aorta/interrupted aortic arch. CT = cor triatriatum. CTOH = conjoined twins with one heart. CVR = congenital vascular ring. DOLV = double-outlet left ventricle. DORV = double-outlet right ventricle. EA = Ebstein's anomaly. EC = ectopia cordis. EFE = endocardial fibroelastosis. HLHS = hypoplastic left heart syndrome. HRHS = hypoplastic right heart syndrome. HS = heterotaxy syndrome. MA = mitral atresia. MVS = mitral valve stenosis. PA = pulmonary atresia. PCDA = premature closure of the ductus arteriosus. PLSVC = persistent left superior vena cava. PS = pulmonary stenosis. PTA = persistent truncus arteriosus. RAA = right aortic arch. SV = single ventricle. TA = tricuspid atresia. TAPVD = total anomalous pulmonary venous drainage. TGA = transposition of the great arteries. TOF = tetralogy of Fallot. TVS = tricuspid valve stenosis. VA = ventricular aneurysm. VSD = ventricular septal defect

### Rare anomaly of fetal CHD

In this study, we defined rare anomalies (RAs) as CHDs whose frequency is less than 1%, as presented in Table 1b. Among all fetal CHDs, 17 subtypes, including five subtypes of fetal CHD—total anomalous pulmonary venous drainage (0·44%), double-outlet left ventricle (DOLV) (0·18%), aortic atresia (AA) (0·14%), aortopulmonary window (0·13%), and ectopia cordis (0·13%)—among major anomalies, and 11 minor anomalies—Ebstein’s anomaly (0·69%), tricuspid atresia (TA) (0·22%), mitral valve stenosis (0·18%), coronary artery fistula (CAF) (0·13%), conjoined twins with one heart (CTOH) (0·09%), mitral atresia (MA) (0·08%), cor triatriatum (CT) (0·07%), ventricular aneurysm (VA) (0·06%), endocardial fibroelastosis (EFE) (0·06%), premature closure of the ductus arteriosus (PCDA) (0·04%), and cardiac diverticulum (CD) (0·02%)—were RAs. These RAs account for 3·44% (1·78% major anomalies and 1·66% minor anomalies) of CHD cases. Furthermore, seven subtypes (CTOH, MA, CT, VA, EFE, PCDA, and CD) whose frequencies were less than 0·1% may be classified as very rare anomalies (vRAs).

### Fetal CHD accompanied with extracardiac defects

**Fig. 3:**
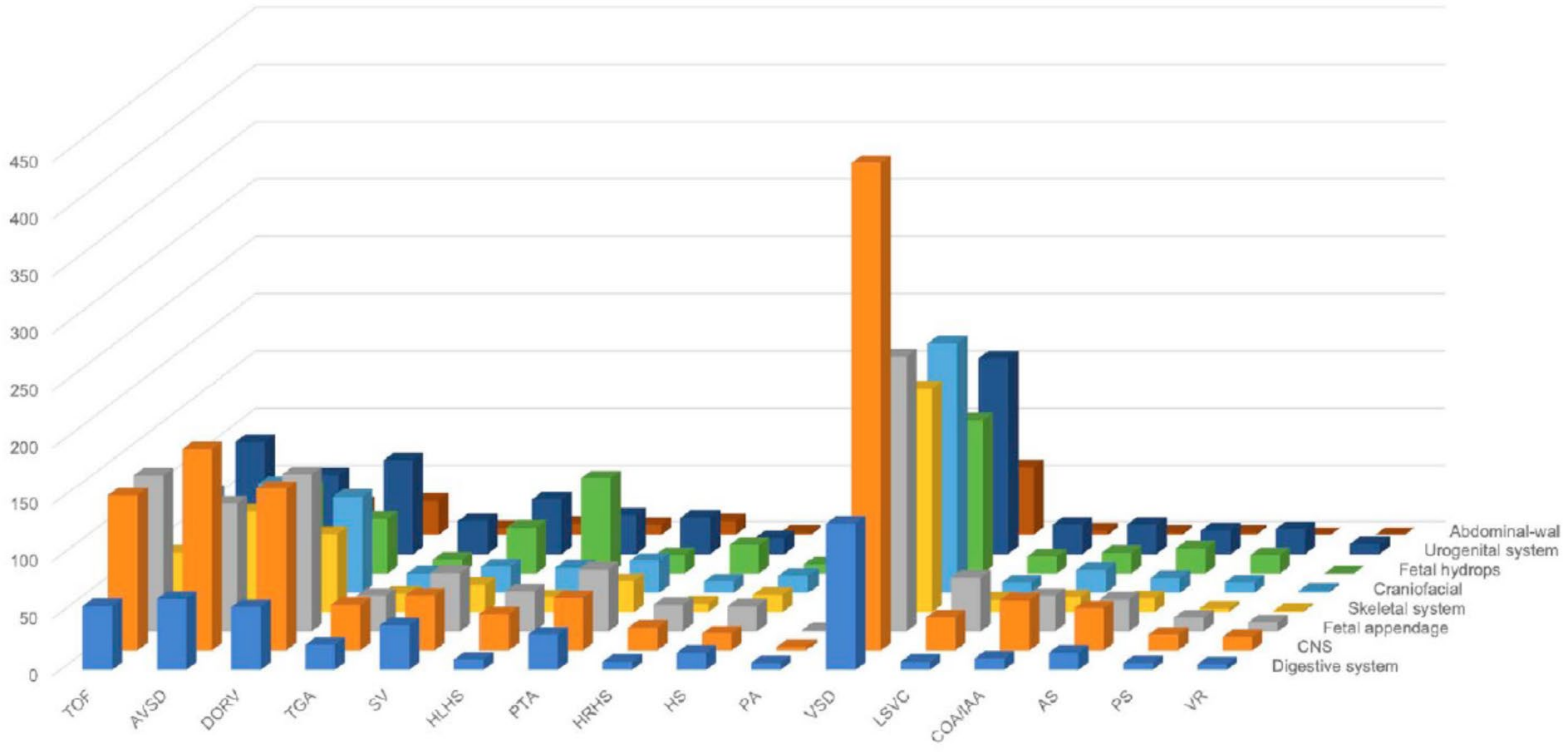
**Extracardiac abnormalities associated with fetal intracardiac anomaly**. The CNS was determined to be the most common extracardiac tissue that accompanies fetal CHD. AS, aortic stenosis. AVSD, atrioventricular septal defect. COA/IAA, coarctation of the aortic arch/interrupted aortic arch. DORV, double-outlet right ventricle. HLHS, hypoplastic left heart syndrome. HRHS, hypoplastic right heart syndrome. HS, heterotaxy syndrome. LSVC, left superior vena cava. PA, pulmonary atresia. PTA, persistent truncus arteriosus. PS, pulmonary stenosis. SV, single ventricle. TGA, transposition of the great arteries. TOF, tetralogy of Fallot. VR, vascular ring. VSD, ventricular septal defect

A total of 5,338 cases of fetal CHD were found to be accompanied by extracardiac malformation(s) and were labeled as extracardiac congenital heart defects (xCHDs). Sixteen subtypes of fetal CHD were identified and correlated with extracardiac malformations (Table 2). VSD was the most common xCHD, accounting for 30% (1,578/5,338) of total xCHDs. AVSD, TOF, and DORV were the next three most common xCHDs, accounting for 13·23% (706/5,338), 12·20% (651/5,338), and 12·14% (648/5,338), respectively. VSD accompanied with central nervous system (CNS) malformation was the most common complex birth defect involved in xCHD, as shown in Fig. 3, accounting for 8% of fetal CHD cases with xCHD (427/5,338). Among all extracardiac malformations, the CNS was the most common organ/tissue to accompany xCHD, accounting for 22·89% (1,222/5,338) of xCHD cases.

**Table 2:** Extracardiac defects associated with congenital heart defects

Disease	TOF	AVSD	DORV	TGA	SV	HLHS	PTA	HRHS	HS	PA	VSD	LSVC	COA/IAA	AS	PS	VR	Total number
**Digestive system malformations**	**56**	**62**	**55**	**22**	**39**	**9**	**31**	**7**	**15**	**6**	**128**	**7**	**10**	**15**	**6**	**5**	**473**
Esophageal atresia	14	17	15	5	10	2	14		8		36	1	3	6	1	1	133
Duodenal atresia/stenosis	15	24	14	3	3		7	1	4	1	41	3	4	2		2	124
Persistent right umbilical vein	12	12	16	8	22	5	8	1	2	4	22		2	3	1	2	120
Hyperechoic bowel	7	5	8	4	3	1	1	5	1	1	18	3	1	4	4		66
Bowel dilatation	8	4	2	2	1	1	1				11						30
**CNS malformations**	**136**	**177**	**142**	**40**	**48**	**32**	**46**	**20**	**15**	**3**	**427**	**29**	**44**	**37**	**14**	**12**	**1222**
Dandy-walker	25	37	21	10	6	8	12	5	3	1	58	2	10	7	2	3	210
Choroid plexus cysts	9	42	35	1	4	1	2	1	3		92	3	3	4	1		201
Medulla oblongata broadening	19	16	18	10	7	6	4	3			77	3	7	7	2	2	181
Lateral ventricle broadening	17	26	14	4	8	5	4	5	3	2	52	11	6	6	3	5	171
Hydrocephalus	16	11	16	5	3	1	9	3	1		34	1	1	4	2	1	108
Holoprosencephaly	19	19	16	3	13	6	7		3		34	4	7	3		1	135
Agenesis of corpus callosum	7	13	6	2	1	2	1		2		23	3	4	4	2		70
Spina bifida	10	7	7	4	4	2	5	2			21		4				66
Arachnoid cyst	8	1	4	1		1					13	2	2		2		34
Spinal meningocele	5	3	3		1		2	1			11						26
Septum pellucidum broadening	1	2	2		1						12			2			20
**Fetal appendage malformations**	**137**	**112**	**138**	**31**	**51**	**35**	**54**	**23**	**22**	**1**	**241**	**47**	**31**	**28**	**12**	**8**	**971**
Single umbilical artery	128	103	113	30	48	34	47	21	22	1	205	47	28	26	11	8	872
Cyst of cord	9	9	25	1	3	1	7	2			36		3	2	1		99
**Skeletal system malformations**	**52**	**88**	**68**	**16**	**24**	**13**	**27**	**7**	**15**	**1**	**196**	**11**	**13**	**12**	**3**	**1**	**547**
Abnormal handing posture	8	23	16	2	3	2	5	1	3		61	1	5	7			137
Talipes equinovarus	16	27	18	7	8	4	5		4		50	3	4		1		147
Absent radius	16	29	21	4	4	3	6	1	3		50	1		3			141
Scoliosis	4	2	6	2	6	2	5		3	1	15	1		1	1		49
Hemipyramid	5	2	2	1	3		4	1			10	5	1		1	1	36
Achondroplasia	3	5	5			2	2	4	2		10		3	1			37
**Craniofacial anomalies**	**88**	**96**	**84**	**17**	**23**	**22**	**28**	**10**	**15**	**4**	**219**	**9**	**20**	**13**	**9**	**2**	**659**
Labial fold	43	32	38	12	9	13	16	3	4	2	89	4	12	9	5	1	292
Nasal bone agenesis	17	30	9	2	4	5	7	2	4	1	57	1	1	3	1		144
Micrognathia	9	19	15	1	5		4	2	2	1	32	1	5		1		97
Eye span long/short	9	10	14	1	3	3	1	3	2		19	3	1	1	2	1	73
Ear deformity	10	5	8	1	2	1			3		22		1				53
**Fetal hydrops**	**54**	**74**	**48**	**12**	**40**	**84**	**16**	**25**	**8**	**7**	**135**	**15**	**18**	**22**	**16**		**574**
Skin dropsy / Separate cystic hygroma	25	36	23	5	16	41	7	7	3	1	64	5	7	10	6	0	256
Hydrothorax/Ascites	29	38	25	7	24	43	9	18	5	6	71	10	11	12	10	0	318
**Urogenital system malformations**	**99**	**70**	**83**	**29**	**48**	**34**	**32**	**14**	**12**	**1**	**172**	**26**	**26**	**21**	**22**	**9**	**698**
Hydronephrosis	22	16	15	7	9	11	4	2	1		31	6	8	4	4	3	143
Polycystic kidney	18	12	19	1	11	5	9	5	2		33	4	3	5	6	2	135
Renal agenesis	25	9	15	3	5	3	7	3			38	8	1	8	7		132
Horseshoe kidney	7	3	6			5	1		1	1	18	3	3	1	1		50
Duplex kidney	3	4	3		2	2					4	3	2	1	2	2	28
Diaphragmatic hernia	15	18	16	11	18	3	9	2	4		35	1	4	2		1	139
Pulmonary hypoplasia	8	5	5	6		5	1	2	2		9		3		2		48
Congenital cystic adenomatoid malformation	1	3	4	1	3	0	1		2		4	1	2			1	23
**Abdominal-wall defects**	**29**	**27**	**30**	**6**	**10**	**9**	**12**	**2**	**1**		**60**	**4**	**2**	**2**			**194**
Omphalocele	27	23	23	4	7	9	11	1	1		52	3	2	2			165
Gastroschisis	2	4	7	2	3		1	1			8	1					29
**Total (n = 5,338)**	**651**	**706**	**648**	**173**	**283**	**238**	**246**	**108**	**103**	**23**	**1,578**	**148**	**164**	**150**	**82**	**37**	**5,338**

AS = aortic stenosis. AVSD = atrioventricular septal defect. COA/IAA = coarctation of the aorta/interrupted aortic arch. DORV = double-outlet right ventricle. HLHS = hypoplastic left heart syndrome. HRHS = hypoplastic right heart syndrome. HS = heterotaxy syndrome. LSVC = left superior vena cava. PA = pulmonary atresia. PS = pulmonary stenosis. PTA = persistent truncus arteriosus. SV = single ventricle. TGA = transposition of the great arteries. TOF = tetralogy of Fallot. VR = vascular ring. VSD = ventricular septal defect

### Genetic study of chromosomal abnormalities with karyotyping and microarray

**Fig. 4:**
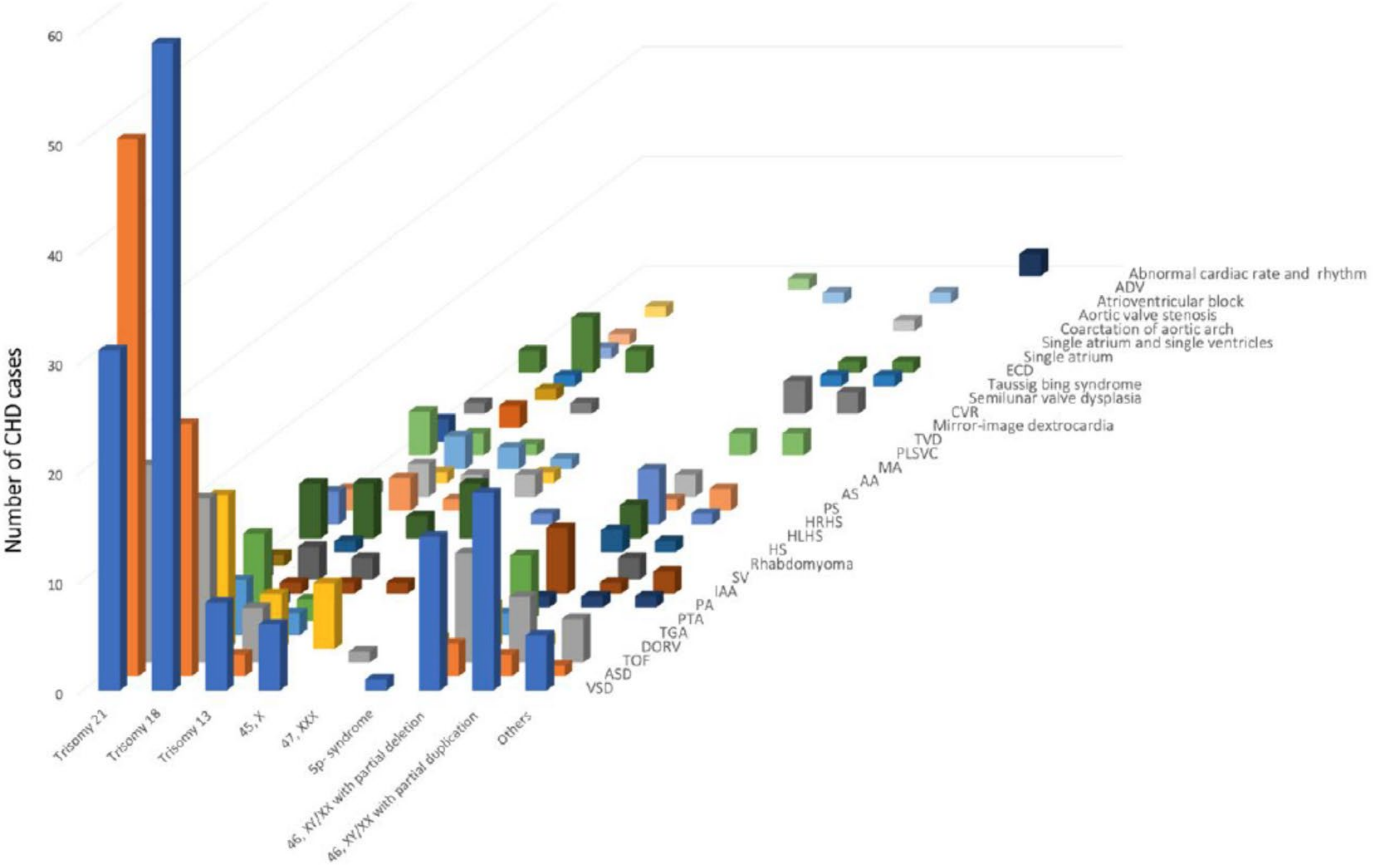
**Karyotyping of fetal CHD**. Trisomy 18 was identified as the most common chromosomal abnormality in fetal CHD, followed by trisomy 21. AA, aortic atresia. ADV, . AS, aortic stenosis. ASD, atrial septal defect. CVR, congenital vascular ring. DORV, double-outlet right ventricle. ECD, endsystolic dimension. HLHS, hypoplastic left heart syndrome. HRHS, hypoplastic right heart syndrome. HS, heterotaxy syndrome. IAA, interrupted aortic arch. MA, mitral atresia. PA, pulmonary atresia. PLSVC, persistent left superior vena cava. PS, pulmonary stenosis. PTA, persistent truncus arteriosus. SV, single ventricle. TGA, transposition of the great arteries. TOF, tetralogy of Fallot. TVD, tricuspid valve disease. VSD, ventricular septal defect

Genetic study for chromosomal abnormality was performed among 566 cases of fetal CHD, 487 of which were analyzed by karyotyping, and 79 by microarray to determine chromosomal copy number variations (CNVs) including DNA fragment deletion and/or repetition/ duplication or uniparental disomy (UPD). As presented in Fig. 4; Table 3, the most common chromosomal abnormality in CHD is trisomy 18 (159/487, 32·65%), followed by trisomy 21 (138/487, 28·34%). When microarray was applied to identify chromosomal segment abnormalities (Table 4), 78·48% (62/79) of cases were found to carry pathogenic microdeletion, microrepetition/microduplication, UPD, or large-segment deletion or repetition. Twenty-two cases were the result of microdeletion, and three of microduplication of chromosome 22q. Twenty-five (of 62) CNVs, including seven in VSD, two in DORV, three in TOF, one each in AS, COA/IAA, hypoplastic right heart syndrome (HRHS), PLSVC, and PTA as well as six in complex CHDs involved in the chromosomal region 22q11 and two (HRHS, TOF) in 22q13, accounted for 40·32% of pathogenic CNVs. The remaining 17 CNVs were considered suspected pathogenic alleles, in which chromosomal deletion 16p11-12 was the predominant abnormality, accounting for 29·41% (5/17) of CNVs.

**Table 3:** Genetic study of Congenital heart defects: Karyotyping of chromosomal anomalies among congenital heart defect subtypes

	Chromosomal anomalies(n = 87)									
Subtype	Trisomy 21 (%)	Trisomy 18 (%)	Trisomy 13 (%)	45, XO (%)	47, XXX (%)	5p- Syndrome (%)	46, XY/XX, with partial deletion (%)	46, XY/XX, with partial duplication (%)	Others (%)	Subtotal (%)
VSD	31 (22·46)	59 (37·11)	8 (21·05)	6 (26·09)		1 (50)	14 (20·59)	18 (41·86)	5 (38·46)	142 (29·16)
ASD	49 (35·51)	23 (14·47)	2 (5·26)				3 (4·41)	2 (4·65)	1 (7·69)	80 (16·43)
TOF	18 (13·04)	15 (9·43)	5 (13·16)		1 (33·33)		10 (14·71)	6 (13·95)	4 (30·77)	59 (12·11)
DORV	7 (5·07)	14 (8·81)	5 (13·16)	6 (26·09)		1 (50)	4 (5·88)	1 (2·33)		38 (7·80)
TGA	4 (2·9)	5 (3·14)	2 (5·26)				2 (2·94)			13 (2·67)
PTA	4(2·9)	8(5·03)	2 (5·26)				6 (8·82)			20 (4·11)
PA	1 (0·72)	1 (0·63)					1 (1·47)	1 (2·33)	1 (7·69)	5 (1·03)
IAA	1 (0·72)	1 (0·63)	1 (2·63)	1 (4·35)			6 (8·82)	1 (2·33)	2 (15·38)	13 (2·67)
SV	2 (1·45)	3 (1·89)	2 (5·26)					2 (4·65)		9 (1·85)
Rhabdomyoma	1 (0·72)									1 (0·21)
HS		1 (0·63)					2 (2·94)	1 (2·33)		4 (0·82)
HLHS	5 (3·62)	5 (3·14)	2 (5·26)	5 (21·74)			3 (4·41)			20 (4·10)
HRHS	3 (2·17)				1(33·33)		5 (7·35)	1 (2·33)		10 (2·05)
PS	2 (1·45)	3(1·89)	1(2·63)				1(1·47)	2(4·65)		9 (1·85)
AS	1 (0·72)	3 (1·89)	2 (5·26)	2 (8·7)			2 (2·94)			10 (2·05)
AA		1 (0·63)		1 (4·35)						2 (0·41)
MA		3 (1·89)	2 (5·26)	1 (4·35)						6 (1·23)
PLSVC	4 (2·9)	2 (1·26)	1 (2·63)				2 (2·94)	2 (4·65)		11 (2·26)
TVD	2 (1·45)									2 (0·41)
MID		2 (1·26)								2 (0·41)
CVR	1 (0.72)		1 (2.63)				3 (4.41)	2 (4·65)		7 (1·44)
SVD		1 (0·63)								1 (0·21)
TBS		1 (0·63)					1 (1·47)	1 (2·33)		3 (0·62)
ECD	2 (1·45)	5 (3·14)	2 (5·26)				1 (1·47)	1 (2·33)		11 (2·26)
SA		1 (0·63)								1 (0·21)
SA-SV		1 (0·63)								1 (0·21)
CAA							1 (1·47)			1 (0·21)
VS		1 (0·63)								1 (0·21)
AB					1 (33·33)		1 (1·47)			2 (0·41)
ADV				1 (4·35)						1 (0·21)
ACRR								2 (4·65)		2 (0·41)
Total	138 (28·34)	159 (32·65)	38 (7·80)	23 (4·72)	3 (0·62)	2 (0·41)	68 (13·96)	43 (8·83)	13 (2·67)	487

AA = aortic atresia. AB = atrioventricular block. ACRR = abnormal cardiac rate and rhythm. ADV = absence of ductus venosus. AS = aortic stenosis. ASD = atrial septal defect. AVS = aortic valve stenosis=. CAA = coarctation of aortic arch. CVR = congenital vascular ring. DORV = double-outlet right ventricle. ECD = endocardial cushion defects. HLHS = hypoplastic left heart syndrome. HRHS = hypoplastic right heart syndrome. HS = heterotaxy syndrome. IAA = interrupted aortic arch. MA = mitral atresia. MID = mirror-image dextrocardia. PA = pulmonary atresia. PLSVC = persistent left superior vena cava. PS = pulmonary stenosis. PTA = persistent truncus arteriosus. SA = single atrium. SV = single ventricle. SVD = semilunar valve dysplasia. TBS = Taussig Bing syndrome. TGA = transposition of the great arteries. TOF = tetralogy of Fallot. TVD = tricuspid valve dysplasia. VSD = ventricular septal defect

**Table 4:** Genetic study of congenital heart defects: Copy number variations of CHD

	Congenital heart defect	Copy number variation(s)	Genotype(s)	Clinical significance	Chromosome(s) involved
1	AS	46, XY, del (22q11.21)(2.1M)	Microdeletion	Pathogenic	Chr. 22q
2	AVSD	46, XX, dup (16q21q22.2)(7.2M)	Microrepetition	Pathogenic	Chr. 16q
3	AVSD	46,XX, del (10q21.3,68.2–68.4,149k) CTNNA3	Microdeletion	Pathogenic	Chr. 10q
4	COA/IAA	46, XX, dup (16p11.2)(0.7M)	Microrepetition	Pathogenic	Chr. 16p
5	COA/IAA	46, XY, dup (16p13.3p13.11)(14.8M)	Microrepetition	Pathogenic	Chr. 16p
6	COA/IAA	46, XX, del (7q33q36.3)(21.5M)	Microdeletion	Pathogenic	Chr. 7q
7	COA/IAA	46, XX, del (22q11.21)(1.4M)	Microdeletion	Pathogenic	Chr. 22q
8	DORV	46,XX, del (22q11.2,2.58M)	Microdeletion	Pathogenic	Chr. 22q
9	DORV	46,XX, del (22q11.21,2.56M),del(6p21.1-12.3,666k)	Microdeletion	Pathogenic	Chr. 22q
10	DORV	46,XX, del (7q11.23,72.3–74.1, 1.7M)	Microdeletion	Pathogenic	Chr. 7q
11	HRHS	46,XY, dup (7q35q36.3,144.4-159.1,14.6M),del(1p36.33p36.32,0.7–4.1,3.3M)	Microrepetition	Pathogenic	Chr. 7q
12	HRHS	46, XX, del (3q29)(1.2M)	Microdeletion	Pathogenic	Chr. 3q
13	HRHS	46, XY, del (3q11.2q12.3)(4.4M)	Microdeletion	uncertainty	Chr. 3q
14	HRHS	46,XY, del (22q13.33,49-51.1,2.1M)	Microdeletion	Pathogenic	Chr. 22q
15	HRHS	46,XY, del (22q11.21,980KB)	Microdeletion	Pathogenic	Chr. 22q
16	HRHS	46,XY, del (7q36.1q36.3).seq[GRCh37/hg19](148287830–159128557)X1	Microdeletion	Pathogenic	Chr. 7q
17	HRHS	46, XX, del (5p14.1p15.33)(28.8M)	Microdeletion	Pathogenic	Chr. 5p
18	PLSVC	46,XY, dup (12p13.31-ter,8.9M),dup(13q31.1-12.11,61M)	Microrepetition	Pathogenic	Chr. 12p
19	PLSVC	46, XY, dup (22q11.21)(2.6M)	Microrepetition	Pathogenic	Chr. 22q
20	PLSVC	46, XY, del (8p23.1)(4.2M)	Microdeletion	Pathogenic	Chr. 8p
21	PTA	46, XX, dup (16p13.11)(1.2M)	Microrepetition	Pathogenic	Chr. 22q
22	PTA	46, XY, dup (22q11.21)(3M)	Microrepetition	Pathogenic	Chr. 16p
23	PTA	46, XY, del (22q11.21)(2.7M)	Microdeletion	Pathogenic	Chr. 22q
24	SV	46,XX, del (3p25.3p26.3).seq[GRCh37/hg19](60064-10330214)X1	Large segment deletion	Pathogenic	Chr. 3p
25	TBS	46, XX, dup (2q11.1q12.3)(14.8M)	Microrepetition	Pathogenic	Chr. 2q
26	TBS, ECD	46, XY, dup (6q23.3q27)(32.8M)	Microrepetition	Pathogenic	Chr. 6q
27	TOF	46, XX, del (8p23.1p23.3)(7.1M)	Microrepetition	Pathogenic	Chr. 8p
28	TOF	46,XX, del (11q24.1-ter,12.4M),dup(15q26.3,3.3M)	Microdeletion	Pathogenic	Chr. 11q
29	TOF	46,XY, del (3q11.2q12.3).seq[GRCh37/hg19](98110933–102528219)X1, 46,XY, del (22q13.2q13.33).seq[GRCh37/hg19](44151871–51225558)X1	Microdeletion	Pathogenic	Chr. 22q and 3q
30	TOF	46, XY, del (22q13.2q13.33)(7.1M)	Microdeletion	Pathogenic	Chr. 22q
31	TOF	46, XY, del (22q11.21)(1.4M), 46, XY, del (22q11.21)(2.6M)	Microdeletion	Pathogenic	Chr. 22q
32	TOF	46, XX, del (22q11.21)(1.4M), del (22q11.21)(0.8M), 46, XY, dup (3p26.3p14.3)(57.7M)	Microdeletion, Microrepetition	Pathogenic	Chr. 22q and 3p
33	TOF	46,XY, dup (3q21.3q27.1).seq[GRCh37/hg19](128583592–184371766)X3	Large segment repetition	Pathogenic	Chr. 3q
34	VSD	46,XY, dup (12p13.31-ter,8.9M),dup(13q31.1-12.11,61M)	Microrepetition	Pathogenic	Chr. 12p
35	VSD	46,XY, dup (22q11.21q11.22,18.6–21.4, 2.8M)	Microrepetition	Pathogenic	Chr. 22q
36	VSD	46, XY, dup (2p23.2p25.3)(28.2M)	Microrepetition	Pathogenic	Chr. 2p
37	VSD	46, XY, dup (16p13.11p13.12)(2M)	Microrepetition	Pathogenic	Chr. 16p
38	VSD	46,XX, del (22q11.21).seq[GRCh37/hg19](18887652–19009027)X1	Pathogenic	Pathogenic	Chr. 22q
39	VSD	46,XY, del (10q21.3,68.2–68.5, 272k)CTNNA3,del(16p12.2,141k)	Pathogenic	Pathogenic	Chr. 10q
40	VSD	46,XX, del (22q11.21,200k)	Microdeletion	Pathogenic	Chr. 22q
41	VSD	46,XY, del (22)(q11.21q11.22,2.56M)	Pathogenic	Pathogenic	Chr. 22q
42	VSD	46, XY, del (13q31.1q34)(29.5M)	Pathogenic	Pathogenic	Chr. 13q
43	VSD	46,XY, del (22q11.2,18.89–20.3,1.4M)	Microdeletion	Pathogenic	Chr. 22q
44	VSD	46,XY, del (22q11.21,2.95M),UPD(11p11.2p11.11,3.4M), 46,XY, del (1p36.21p36.33).seq[GRCh37/hg19](823534-15632453)X1	Microdeletion	Pathogenic	Chr. 22q and 1p
45	VSD	46,XX, dup (12p11.1p13.33).seq[GRCh37/hg19](60105-34812049)X3	Large segment repetition	Pathogenic	Chr. 12p
46	VSD	46,XY, dup (3p26.3p14.3).seq[GRCh37/hg19](60064-57755328)X3	Large segment repetition	Pathogenic	Chr. 3p
47	ACRR	46,XY, dup (22q11.21q11.22,18.6–21.4, 2.8M)	Microrepetition	Pathogenic	Chr. 22q
48	DORV, PS, Dextrocardia	46,XX, del (22q11.21,18.8–21.4,1.21M)	Microdeletion	Pathogenic	Chr. 22q
49	HRHS, TVS, VSD	46,XX, del (17)(p13.3, 0.018–2.63,2.61M),dup(4)(q13.1,63.8–65,1.2M)	Microdeletion Microrepetition	Pathogenic	Chr. 17p and 4q
50	IAA, VSD, PLSVC	46,XX, dup(8p12-ter,32.7M),del(5p15.33,3.2M)	Large segment repetition	Pathogenic	Chr. 8p and 5p
51	IAA, VSD, PLSVC	46,XX,del (7q33q36.3).seq[GRCh37/hg19](137529688–159068966)X1	Large segment deletion	Pathogenic	Chr. 7q
52	TGA, RAA	46, XY, dup (14q31.3q32.33)(20.4M)	Microrepetition	Pathogenic	Chr. 14p
53	TGA, RAA	46, XX, del (22q11.21)(1.7M)	Microdeletion	Pathogenic	Chr. 22q
54	TOF, PA	46, XY, del (22q11.21)(2.7M)	Microdeletion	Pathogenic	Chr. 22q
55	TOF, PA	46, XY, dup (3q21.3q26.1)(33.9M), dup (3q26.1q27.1)(21.8M)	Microrepetition	Pathogenic	Chr. 3q
56	VR (RDA)	46, XX, dup (10q11.21q24.32)(61.3M)	Microrepetition	Pathogenic	Chr. 10q
57	VR(ALSA)	46, XX, del (3p25.3p26.3)(10.3M)	Microdeletion	Pathogenic	Chr. 3p
58	VR(ALSA)	46, XX, del (22q11.21)(3M)	Microdeletion	Pathogenic	Chr. 22q
59	VR(ALSA)	46, XX, del (22q11.21)(1.4M)	Microdeletion	Pathogenic	Chr. 22q
60	VR(DAA)	46, XX, del (5p14.3p15.33)(19.5M)	Microdeletion	Pathogenic	Chr. 5p
61	VSD, PA	46, XY, del (1p36.21p36.33)(14.8M)	Microdeletion	Pathogenic	Chr. 1p
62	VSD, PLSVC	46,XY, del (22q11.21).seq[GRCh37/hg19](18920346–21601628)X1, 46, XX, dup (12p11.1p13.33)(34.8M)	Large segment deletion, Microrepetition	Pathogenic	Chr. 22q
63	Atrioventricular block	46,XX, dup (15q11.2,20.1–23, 2.93M)	Microrepetition	Suspected pathogenic	Chr. 15q
64	DORV	46,XY, dup (16p11.1p11.2,4.0M) CTF1	Uniparental disomy	Suspected pathogenic	Chr. 16p
65	HS	46,XY, dup (3p26.3,0.9–1.4,488K)	Microrepetition	Suspected pathogenic	Chr. 3p
66	PS	46,XY, dup (1q21.2-21.3,148-149.7,1.728M)	Microrepetition	Suspected pathogenic	Chr. 1q
67	TBS, ECD	46, XX, del (16p11.2)(0.7M)	Microdeletion	Suspected pathogenic	Chr. 16p
68	TOF	46, XX, dup (Xp22.2)(0.2M)	Microrepetition	Suspected pathogenic	Chr. Xp
69	TOF	46,XX, dup (6q25.1-25.3,151.5-158.1,6.6M)	Microrepetition	Suspected pathogenic	Chr. 6q
70	TOF	46,XY, dup (6q23.3q27).seq[GRCh37/hg19](138052325–170879606)X3	Large segment deletion, Microrepetition	Suspected pathogenic	Chr. 6q
71	TOF	46, XX, del (22q11.21)(0.1M)	Microdeletion	Suspected pathogenic	Chr. 22q
72	TOF(PA)	46, XY, del (16p13.3)(0.1M), del (22q11.21)(2.6M)	Microdeletion	Suspected pathogenic	Chr. 16p
73	VSD	46,XY, dup (1q21.2-21.3,148-149.7,1.728M)	Microrepetition	Suspected pathogenic	Chr. 1q
74	VSD	46,XX, dup (15q11.2,20.1–23, 2.93M)	Microrepetition	Suspected pathogenic	Chr. 15q
75	VSD	46,XY, dup (2p25.3p23.2),seq[GRCh37/hg19](10001-28278298)X3	Microrepetition	Suspected pathogenic	Chr. 2p
76	VSD	46, XX, del (16p12.2)(0.8M)	Microdeletion	Suspected pathogenic	Chr. 16p
77	VSD	46,XX, dup (1)(p22.1p21.3,92-94.8,2.8M), 46,XY, dup (3p26.3,0.9–1.4,488K)	Microrepetition	Suspected pathogenic	Chr. 1p and 3p
78	VSD, IAA	46,XX, UPD (16p11.1p11.2,4.7M) CTF1	Uniparental disomy	Suspected pathogenic	Chr. 16p
79	ACRR	46,XX, dup (1)(p22.1p21.3,92-94.8,2.8M)	Microrepetition	Suspected pathogenic	Chr. 1p

AB = atrioventricular block. ACRR = abnormal cardiac rate and rhythm. ALSA = aberrant left subclavian artery. AS = aortic stenosis. AVSD = atrioventricular septal defect. COA/IAA = coarctation of the aorta/interrupted aortic arch. DAA = double arch of the aorta. DORV = double-outlet right ventricle. ECD = endocardial cushion defect. HRHS = hypoplastic right heart syndrome. HS = heterotaxy syndrome. IAA = interrupted aortic arch. PA = pulmonary atresia. PLSVC = persistent left superior vena cava. PS = pulmonary stenosis. PTA = persistent truncus arteriosus. RAA = right aortic arch. RDA = right descending aorta. SV = single ventricle. TBS = Taussig Bing syndrome. TGA = transposition of the great arteries. TOF = tetralogy of Fallot. TVS = tricuspid valve stenosis. VR = vascular ring. VSD = ventricular septal defect

### Termination of pregnancy as a consequence of prenatal screening of CHD

**Fig. 5:**
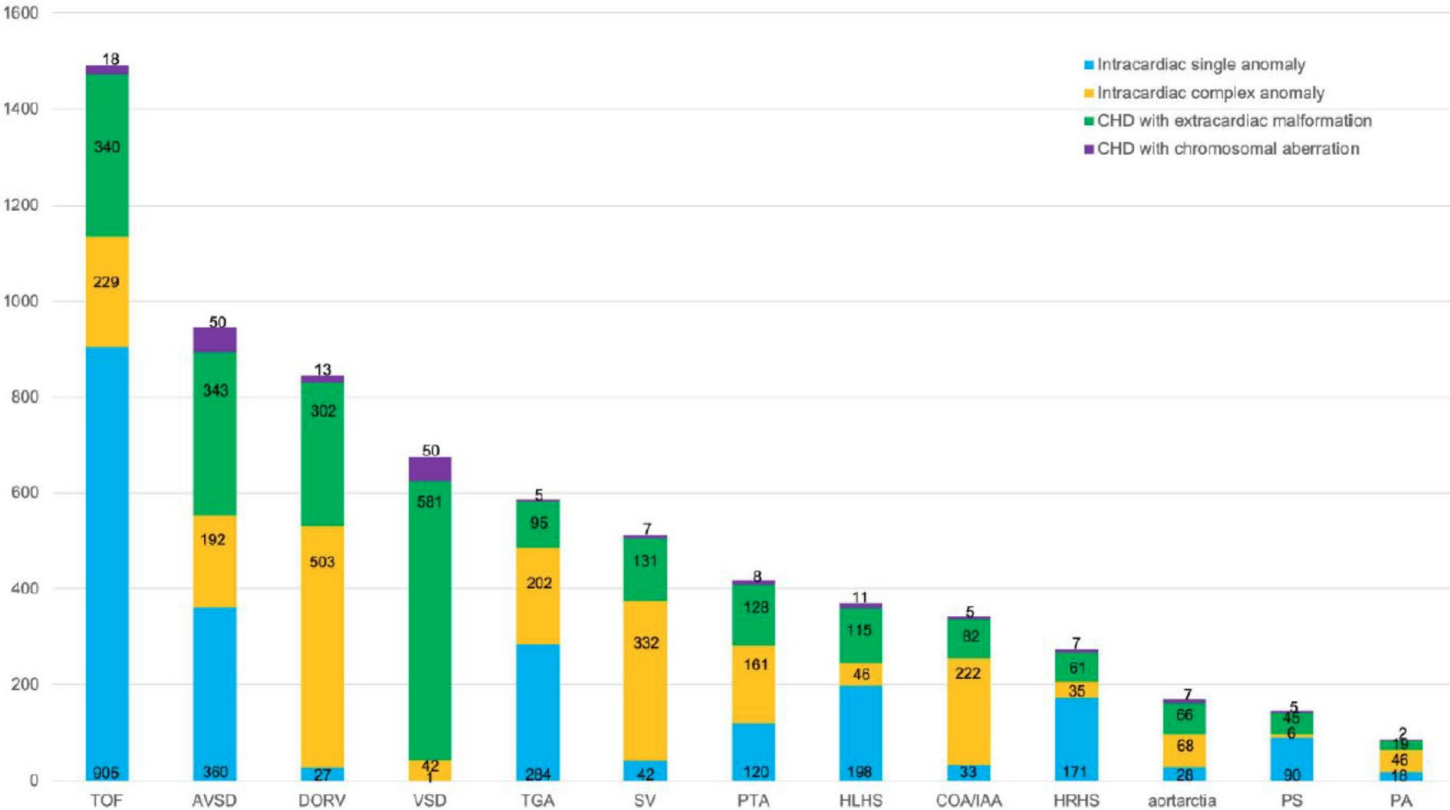
**Comparisons of intracardiac anomalies and extracardiac anomalies**. It is shown that the most common fetal CHD in each subcategory was: TOF in intracardiac single anomaly (light blue), DORV in intracardiac complex anomaly (yellow), and VSD in extracardiac malformation (green). AVSD, atrioventricular septal defect. COA/IAA, coarctation of the aorticarch/interrupted aortic arch. DORV, double-outlet right ventricle. HLHS, hypoplastic left heart syndrome. HRHS, hypoplastic right heart syndrome. PA, pulmonary atresia. PS, pulmonary stenosis. PTA, persistent truncus arteriosus. SV, single ventricle. TGA, transposition of the great arteries. TOF, tetralogy of Fallot. VSD, ventricular septal defect

Among 12,441 CHD cases for which data were available, 60·69% (7,551/12,441) pregnancies were confirmed to have been selectively terminated. Of these aborted fetuses, 6,857 underwent postnatal pathological autopsy, which characterized 2,277 (33·21%) with intracardiac simple CHDs, 2,084 (30·39%) with intracardiac complex CHDs, and 2,308 (33·66%) with CHDs with extracardiac malformation(s), and 188 cases (2·74%) were independently identified as carrying chromosomal abnormalities. TOF was the most common CHD among the aborted fetuses, followed in descending order by AVSD, DORV, VSD, TGA, SV, PTA, HLHS, COA/IAA, and HRHS, as shown in Fig. 5.

### Postnatal follow-up to verify the accuracy of prenatal ultrasound screening results

Prenatal ultrasound is a screening, rather than a diagnostic, procedure. In this study, postnatal follow-up was carried out for 20% (3,619) of 18,171 fetal CHD cases, including 1,965 cases by the postnatal diagnostic procedure(s) of clinical visits, imaging (chest X-ray, ultrasound, and/or MRI), and neonatal surgery; and 1,654 cases by pathological autopsy of aborted fetuses (Table 5). Our follow-up results showed 4·70% of cases with 100% agreement between prenatal screening results and postnatal verification, for the CHD subtypes of AA, CAF, dextrocardia, DOLV, ductus arteriosus stenosis, HLHS, HRHS, heterotaxy syndrome, MA, myocardiopathy, pulmonary atresia/VSD, PCDA, patent ductus arteriosus, pulmonary valvular stenosis, TA, tricuspid valve cleft, and tricuspid valve dysplasia. Other than three subtypes—two (aortic valve stenosis and noncompaction of the ventricular myocardium) that were completely misreported and 44 cases of COA/IAA that showed 45.45% agreement—the majority of the prenatal ultrasound results showed more than 60% agreement between prenatal screening results and postnatal follow-up confirmation. Overall, prenatal ultrasound resulted in 88% accuracy.

**Table 5:** Postnatal follow-up to verify the accuracy of prenatal ultrasound screening

Congenital heart defect	Postnatal agreement with prenatal screening	Postnatal disagreement with prenatal screening	Number of cases followed up	Agreement (%)
Postnatal imaging and surgery				
VSD	738	98	836	88·28%
PLSVC	133	4	137	97·08%
ECD	115	10	125	92·00%
TOF	112	22	134	83·58%
CVR	104	4	108	96·30%
DORV	91	10	101	90·10%
TGA	54	4	58	93·10%
SV	49	11	60	81·67%
PS	44	12	56	78·57%
COA/IAA	20	24	44	45·45%
AS	42	7	49	85·71%
PTA	38	11	49	77·55%
PVS	32	0	32	100·00%
HS	17	0	17	100·00%
ASA	16	6	22	72·73%
Cardiac tumor	14	1	15	93·33%
HLHS	25	3	28	89·29%
EA	10	2	12	83·33%
Dextrocardia	10	0	10	100·00%
HRHS	9	0	9	100·00%
PA/VSD	7	0	7	100·00%
Dextrocardia	7	0	7	100·00%
TA	6	0	6	100·00%
Myocardiopathy	5	0	5	100·00%
DAS	5	0	5	100·00%
HRHS	4	1	5	80·00%
TVD	4	0	4	100·00%
TAPVD	3	1	4	75·00%
CAF	3	0	3	100·00%
CT	3	1	4	75·00%
MA	2	0	2	100·00%
PA	2	1	3	66·67%
AA	2	0	2	100·00%
DOLV	1	0	1	100·00%
PCDA	1	0	1	100·00%
PDA	1	0	1	100·00%
TVC	1	0	1	100·00%
NVM	0	1	1	0·00%
AVS	0	1	1	0·00%
Subtotal	**1 = 730**	**235**	**1,965**	**88·04%**
Autopsy of aborted fetus				
VSD	621	53	674	92·14%
ECD	219	15	234	93·59%
TOF	153	21	174	87·93%
DORV	145	16	161	90·06%
TGA	111	9	120	92·50%
PTA	54	19	73	73·97%
PA	30	19	49	61·22%
COA/IAA	24	30	54	44·44%
SV	29	1	30	96·67%
Cardiac tumor	25	3	28	89·29%
HS	22	0	22	100·00%
HLHS	22	0	22	100·00%
HRHS	13	0	13	100·00%
Subtotal	**1,468**	**186**	**1,654**	**88·75%**
**Total**	**3,198**	**421**	**3,619**	**88·37%**

AA = aortic atresia. AS = aortic stenosis. ASA = atrial septal aneurysm. AVS = aortic valve stenosis. CAF = coronary artery fistula. COA/IAA = coarctation of the aorta/interrupted aortic arch. CT = cor triatriatum. CVR = congenital vascular ring. DAS = ductus arteriosus stenosis. DOLV = double-outlet left ventricle. DORV = double-outlet right ventricle. EA = Ebstein's anomaly. ECD = endocardial cushion defect. HLHS = hypoplastic left heart syndrome. HRHS = hypoplastic right heart syndrome. HS = heterotaxy syndrome. MA = mitral atresia. NVM = noncompaction of the ventricular myocardium. PA = pulmonary atresia. PCDA = premature closure of the ductus arteriosus. PDA = patent ductus arteriosus. PLSVC = persistent left superior vena cava. PS = pulmonary stenosis. PTA = persistent truncus arteriosus. PVS = pulmonary vein stenosis. SV = single ventricle. TA = tricuspid atresia. TAPVD = total anomalous pulmonary venous drainage. TVC = tricuspid valve cleft. TVD = tricuspid valve dysplasia. TGA = transposition of the great arteries. TOF = tetralogy of Fallot. VSD = ventricular septal defect

## Discussion

### Incidence and distribution

Applying prenatal ultrasound screening, we determined that the incidence of CHDs in China during 2011–2013 was 7·4099 per 1,000 (7·4099%) pregnancies. To our knowledge, this is the largest single cohort in the world, with 2,452,249 pregnancies reported so far. The incidence of Chinese fetal CHD cases in the current report is similar to that reported in Western countries (Table S3) [23–26]. The incidence is higher in advanced, developed regions, such as in the southeastern provinces of Shandong, Jiangsu, and Guangdong, where CHD incidence is ≥ 9% (Fig. 1), and in provinces with a high rate of birth defects, such as 9·8% in Shanxi province [22]. CHD incidence is much lower—below 6%—in the western provinces of Yunnan, Qinghai, Gansu, and Xinjian (Fig. 1). However, the higher incidence in coastal areas vs. the low rate in western developing regions appears not to be correlated with economic development (Figure S2).

### Spectrum of CHDs: single vs. complex and intracardiac vs. extracardiac

A total of 36 subtypes of fetal CHD, including 15 major and 21 minor abnormalities, have been reported in this study. To our knowledge, this is the most complete collection of fetal CHD cases among Chinese populations. We determined that the most common single intracardiac anomaly was VSD, followed by TOF, AVSD, PLSVC, and TGA. Among the intracardiac multi-anomalies, DORV was the most common, followed by SV, COA/IAA, TOF, and AVSD. We also documented that the CNS was found to be the most commonly involved extracardiac tissue, accounting for 20·89% of extracardiac defects, followed by the urogenital (15·21%), craniofacial (13·52%), digestive (8·60%), and skeletal (7·99%) systems. Fetal appendage malformations (21·04%), fetal hydrops (8·29%), and fetal abdominal-wall defects (4·45%) were also found to occur commonly with intracardiac anomaly.

### Genetics and genomics: Cytogenetics vs. molecular genetics

**Fig. 6:**
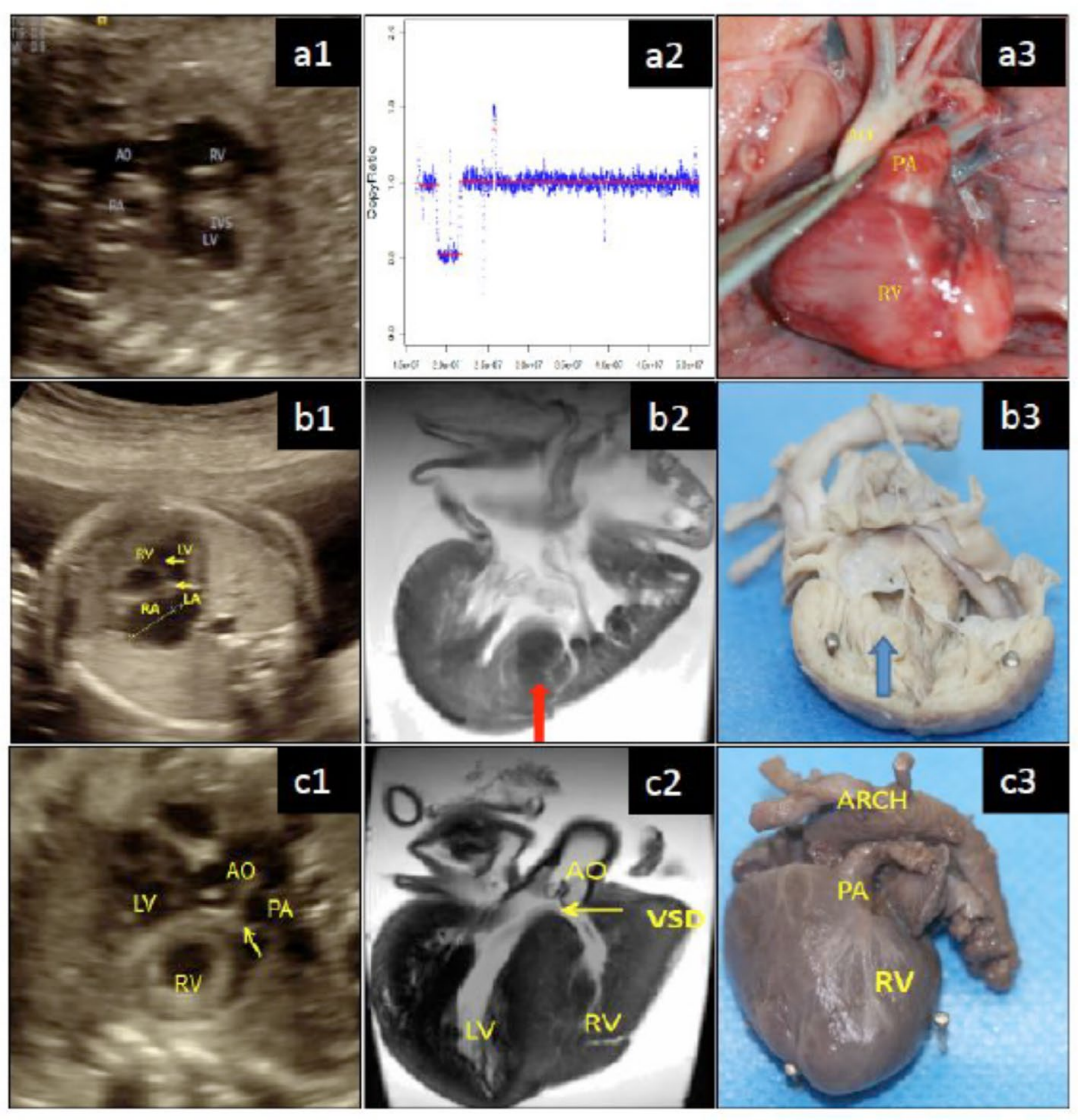
Comprehensive study of CHD. Prenatally identified CHD by fetal echocardiography (a1, b1, c1) was confirmed with MRI (b2, c2), pathological autopsy (a3, b3, c3), and genetic studies of microarray that showed microdeletion (a2) or genome wide sequencing (not shown). Three cases are presented to demonstrate the prenatally fetal-echocardiography-screening were verified and confirmed with MRI, autopsy, and genetic/genomic approach. Case 1 (a): DiGeorge syndrome was observed with DORV by prenatal ultrasound (a1) and verified in pathological autopsy (a3). Genetic study with a microarray (a2) determined a microdeletion of 2,825,77Kb at chromosome 22q11.21 between 22.18920346-21746118, which fell into the DiGeorge critical region (DGCR) that embeds 69 genes. Case 2 (b); CHARGE syndrome was observed with AVSD by prenatal ultrasound (b1) and verified in pathological autopsy (a3). Genetic study with a microarray (a2), confirmed by a 7T MRI (as pointed by arrow in b2), verified by a single nucleotide deletion c.6482del at gene CHD7 (NM_017780.3), which resulted in a frameshift mutation p.His2161Leufs54 and produced a mutant C-terminal peptide of CHD7 protein. Case 3 (c): Noonan syndrome (type X) was identified with a TOF presented by VSD, PA, and ARCH in the echocardiography (c1), which was confirmed by a 7T MRI (c2) and pathological autopsy (c3). GWS determined a single nucleotide mutation c.309C>A at gene LZTR1 (NM_006767.3), resulting in a nonsense mutation of p.Cyc103* that truncated C-terminus of LZTR1 protein

It has long been known that CHDs may result from chromosomal abnormalities. CHD in Down syndrome (DS, trisomy 21) and in DiGeorge syndrome, characterized by microdeletion of the DiGeorge critical region at 22q11.21, represents two traditional conditions [7, 8]. DS is by far the most common and the best-known disorder resulting from chromosomal aneuploidy and the most common cause of intellectual disability. CHD is the leading cause of mortality and morbidity during the first two years of life in the DS population [27]. DiGeorge syndrome is caused by the deletion of a small segment of chromosome 22 [28]. Advanced genetic studies have identified about 400 genes implicated in CHDs, encompassing transcription factors, cell signaling molecules, and structural proteins that are important for heart development [29]. In our study, we initiated chromosomal karyotyping to determine the aneuploidy, followed by microarray assay for CNVs (Tables 3 and 4), to identify chromosomal abnormalities among the fetal CHD cases identified through prenatal echocardiography. In this study, four cases of TOF showed micro-deletion at 22q11.21, which is characteristic of DiGeorge syndrome. Micro-deletion at this DiGeorge’s locus was also the most common abnormality in 53·84% of VSD cases. Genome-wide association study (GWS) was also performed, as presented in Fig. 6. One case was CHARGE syndrome, which was originally screened as AVSD with prenatal fetal echocardiography, diagnosed with MRI, confirmed by autopsy, and studied with GWS, which identified a c.6482del at gene *CHD7* that produced a mutant peptide. The second example was a point mutation, c.309 C > A, at gene *LZTR1*, identified from a case of fetal TOF and determined to be Noonan syndrome.

### Clinical application and data reliability

The overall 88% agreement in this study between prenatal ultrasound results and the follow-up confirmation (Table 5) demonstrates the feasibility and capability of performing a nation-wide study with a standardized procedure. Our experience is that system-wide training in general prenatal ultrasound skills and knowledge with on-site initiation of prenatal ultrasound screening to recognize fetal cardiac anomalies is critical for success. Whether the affected fetus is born or is terminated, postnatal follow-up may determine the accuracy of screening outcome, especially at early stages. This follow-up may help in implementation of prenatal screening for CHD in under-developed regions. In developed areas, we encourage clinicians to consider the comprehensive approach to confirmation of results from prenatal screening for CHD, integrating prenatal ultrasound, MRI, pathological autopsy, postnatal cardiac ultrasound or imaging, and genetic studies (Fig. 6), to better understand the molecular pathogenesis of CHD. With this approach, we will be able to explore and implement early intervention to prevent fetal CHD and to reduce the global burden of treatment for CHDs.

### Electronic supplementary material

Below is the link to the electronic supplementary material.

Supplementary Material 1

## Abbreviations

AA: aortic atresia
APW: aortopulmonary window
AVSD: atrioventricular septal defect
CAF: coronary artery fistula
CD: cardiac diverticulum
COA/IAA: coarctation of the aortic arch/interrupted aortic arch
CT: cor triatriatum
DOLV: double-outlet left ventricle
DORV: double-outlet right ventricle
EA: Ebstein’s anomaly
EC: ectopia cordis
EFE: endocardial fibroelastosis
HLHS: hypoplastic left heart syndrome
HRHS: hypoplastic right heart syndrome
MA: mitral atresia
PCDA: premature closure of the ductus arteriosus
PLSVC: persistent left superior vena cava
PTA: persistent truncus arteriosus
SV: single ventricle
TA: tricuspid atresia
TBS: Taussig Bing syndrome
TGA: transposition of the great artery
TOF: tetralogy of Fallot
VA: ventricular aneurysm
VSD: ventricular septal defect

## References

[1] Hoffman JI, Kaplan S (2002). The incidence of congenital Heart Disease. J Am Coll Cardiol.

[2] Wu W, He J, Shao X (2020). Incidence and mortality trend of congenital Heart Disease at the global, regional, and national level, 1990–2017. Med (Baltim).

[3] Yang XY, Li XF, Lü XD, Liu YL (2009). Incidence of congenital Heart Disease in Beijing, China. Chin Med J (Engl).

[4] Zhao QM, Ma XJ, Jia B, Huang GY (2013). Prevalence of congenital Heart Disease at live birth: an accurate assessment by echocardiographic screening. Acta Paediatr.

[5] Zhao QM, Liu F, Wu L, Ma XJ, Niu C, Huang GY (2019). Prevalence of congenital Heart Disease at live birth in China. J Pediatr.

[6] Liu F, Yang YN, Xie X (2015). Prevalence of congenital Heart Disease in Xinjiang multi-ethnic region of China. PLoS ONE.

[7] Zaidi S, Brueckner M (2017). Genetics and genomics of congenital Heart Disease. Circ Res.

[8] Thomford NE, Dzobo K, Yao NA (2018). Genomics and epigenomics of congenital heart defects: Expert review and lessons learned in Africa. OMICS.

[9] Rosa-Garrido M, Chapski DJ, Vondriska TM (2018). Epigenomes in Cardiovascular Disease. Circ Res.

[10] Courtney JA, Cnota JF, Jones HN (2018). The role of abnormal placentation in congenital Heart Disease; cause, correlate, or consequence?. Front Physiol.

[11] Puri K, Warshak CR, Habli MA (2017). Fetal somatic growth trajectory differs by type of congenital Heart Disease. Pediatr Res.

[12] Laas E, Lelong N, Thieulin AC (2012). Preterm birth and congenital heart defects: a population-based study. Pediatrics.

[13] Jorgensen M, McPherson E, Zaleski C, Shivaram P, Cold C (2014). Stillbirth: the heart of the matter. Am J Med Genet A.

[14] Auger N, Fraser WD, Healy-Profitos J, Arbour L (2015). Association between preeclampsia and congenital heart defects. JAMA.

[15] Russo MG, Paladini D, Pacileo G (2008). Changing spectrum and outcome of 705 fetal congenital Heart Disease cases: 12 years, experience in a third-level center. J Cardiovasc Med.

[16] Kaasen A, Tuveng J, Heiberg A, Scott H, Haugen G (2006). Correlation between prenatal ultrasound and autopsy findings: a study of second-trimester abortions. Ultrasound Obstet Gynecol.

[17] Carvalho JS, Allan LD, International Society of Ultrasound in Obstetrics and Gynecology (2013). ISUOG Practice guidelines (updated): Sonographic screening examination of the fetal heart. Ultrasound Obstet Gynecol.

[18] Zhang Y, Riehle-Colarusso T, Correa A (2011). Observed prevalence of congenital heart defects from a surveillance study in China. J Ultrasound Med.

[19] Lees CC, Stampalija T, Baschat A (2020). ISUOG Practice guidelines: diagnosis and management of small-for-gestational-age fetus and fetal growth restriction. Ultrasound Obstet Gynecol.

[20] Fetal Echocardiography Task Force; American Institute of Ultrasound in Medicine Clinical Standards Committee; American College of Obstetricians and Gynecologists; Society for Maternal-Fetal Medicine (2011). AIUM practice guideline for the performance of fetal echocardiography. J Ultrasound Med.

[21] Hedegaard H, Johnson RL (2019). An Updated International Classification of Diseases, 10th revision, clinical modification (ICD-10-CM) Surveillance Case Definition for Injury hospitalizations. Natl Health Stat Report.

[22] Li Z, Ren A, Zhang L (2006). Extremely high prevalence of neural tube defects in a 4-county area in Shanxi Province, China. Birth Defects Res a Clin Mol Teratol.

[23] Reller MD, Strickland MJ, Riehle-Colarusso T, Mahle WT, Correa A (2008). Prevalence of congenital heart defects in metropolitan Atlanta, 1998–2005. J Pediatr.

[24] Fesslova V, Nava S, Villa L (1999). Evolution and long-term outcome in cases with fetal diagnosis of congenital Heart Disease: Italian multicentre study. Fetal Cardiol Study Group Italian Soc Pediatr Cardiol.

[25] Allan LD, Sharland GK, Milburn A (1994). Prospective diagnosis of 1,006 consecutive cases of congenital Heart Disease in the fetus. J Am College Cardiol.

[26] Tennstedt C (1999). Spectrum of congenital heart defects and extracardiac malformations associated with chromosomal abnormalities: results of a seven year necropsy study. Heart.

[27] Levenson D (2009). Talking about Down syndrome. Am J Med Genet A.

[28] DiGeorge AM (1965). Discussions on a new concept of the cellular basis of immunity. J Pediatr.

[29] Williams K, Carson J, Lo C (2019). Genetics of congenital Heart Disease. Biomolecules.

