# Supplementary material for: Incidence, distribution, disease spectrum, and genetic deficits of congenital heart defects in China: implementation of prenatal ultrasound screening identified 18,171 affected fetuses from 2,452,249 pregnancies
Source: Cell Biosci. 2023 Dec 19;13:229. doi: 10.1186/s13578-023-01172-7 (PMC10731863; doi:10.1186/s13578-023-01172-7)
Supplement: Supplementary file 1 — Supplementary Material 1 [file 13578_2023_1172_MOESM1_ESM.docx]

***Table S1:* Chinese consortium for prenatal ultrasound screening for congenital heart defects**

| **Province** | **Ultrasound Specialist** | **Hospital** |
| --- | --- | --- |
| **Hubei** | Wenjun Zhang | Taihe Hospital, Shiyan, Hubei |
|  | Yi Wang | People's Hospital, Macheng, Hubei |
|  | Jun Cheng | Women and Children Hospital, Ezhou, Hubei |
|  | Yuqiong Chen | Women and Children Hospital, Songzi, Hubei |
|  | Ting Lei | Songzi Family Planning Service Center |
|  | Shaowei Zhong | Women and Children Hospital, Shiyan, Hubei |
|  | Qingxiu Ai | The Central Hospital of Enshi Tujia and Miao Autonomous Prefecture, Enshi, Hubei |
|  | Xin Liu | Maternity and Children's Health Hospital, Huangshi, Hubei |
|  | Bin Xie | Huanggang Central Hospital, Huanggang, Hubei |
|  | Yang Lv | Maternal and Child Health Hospital, Xiangyang, Hubei |
|  | Yongfu Shao | Xiangyang Central Hospital, Xiangyang, Hubei |
|  | Zheng Wang | Maternal and Child Health Hospital of Yingshan County, Yingshan, Hubei |
|  | Jiali Cai | Women and Children Hospital, Anlu, Hubei |
|  | Qiang Zhang | Jingzhou Central Hospital, Jingzhou, Hubei |
|  | Wei Xu | The First People's Hospital, Jingzhou, Hubei |
|  | Lihong Wen | Women and Children Hospital, Gongan, Hubei |
|  | Yichao Tao | Xiaogan Central Hospital, Xiaogan, Hubei |
|  | Zheng Ye | Maternal and Child Health Hospital, Xiaogan, Hubei |
|  | Hongyi Ren | Hubei Aerospace Hospital, Xiaogan, Hubei |
|  | Ya Wang-Xuelian Zhu | Zhijiang People's Hospital, Zhijiang, Hubei |
|  | Chuanfen Li | Women and Children Hospital, Yichang, Hubei |
|  | Weiying Ji | Wuhan Maternal and Child Healthcare Hospital, Tongji Medical College, Huazhong University of Science & Technology, Wuhan, Hubei |
|  | Xinlin Chen | Maternal and Child Health Hospital of Hubei Province, Wuhan, Hubei |
| **Fujian** | Shuping Yang | Zhangzhou Municipal Hospital, Zhangzhou, Fujian |
|  | Hong Wang | Fuzhou General Hospital, Fuzhou, Fujian |
| **Shandong** | Yan Xu | Rizhao People's Hospital, Rizhao, Shangdong |
|  | Taotao Chen | Qingdao Women and Children’s Hospital, Qingdao, Shangdong |
| **Guangdong** | Xiaoying Lin | Baoan Women's and Children's Hospital Affiliated to Jinan University, Shenzhen, Guangdong |
|  | Xiaoyan Ma | Women and Children Hospital of Guangdong Provincial, Guangzhou, Guangdong |
|  | Jin Li | Nanshan Maternity and Child Healthcare Hospital, Shenzhen, Guangdong |
| **Tianjin** | Zhikun Zhang | Central Hospital of Gynecology Obstetrics, Tianjin |
| **Beijing** | Xiaoxia Ni | The 306 Hospital, Beijing |
|  | Qingqing Wu | Beijing Obstetrics and Gynecology Hospital Affiliated to Capital Medical University, Beijing |
| **Liaoning** | Chunli Jing | Dalian Maternal and Child Health Care Hospital, Dalian, Liaoning |
|  | Xuezhu Shao | Jinzhou Women and Infant's Hospital, Liaoning |
|  | Yunping Guan | Shenyang Women and Children's Hospital, Shanyang, Liaoning |
| **Jiangsu** | Xuedong Deng | Suzhou Municipal Hospital, Suzhou, Jiangsu |
|  | Tong Ru | Gulou Hospital, Nanjing, Jiangsu |
|  | Li Cao | Nanjing Municipal Maternity and Child Healthcare Hospital, Nanjing, Jiangsu |
| **Guangxi** | Shuihua Yang | The Maternal and Child Healthcare Hospital of Guangxi Zhuang Autonomous Region, Nanning, Guangxi |
| **Zhejiang** | Hong Lu | Women's Hospital Affiliated to Zhejiang University School of Medicine, Hanzhou, Zhejiang |
| **Anhui** | Zhongping Mu | Maternal and Child Health Hospital of Anhui Province, Hefei, Anhui |
|  | Ling Wang | The First Affiliated Hospital of Anhui Medical University, Hefei, Anhui |
|  | Fan Jiang | The Second Affiliated Hospital of Anhui Medical University, Hefei, Anhui |
| **Henan** | Ruili Wang | Henan Provincial People's Hospital, Zhengzhou, Henan |
|  | Hezhou Li | The Third Affiliated Hospital of Zhengzhou University, Zhengzhou, Henan |
| **Hunan** | Hongxia Yuan | Changsha Municipal Hospital for Maternal and Child Healthcare, Changsha, Hunan |
|  | Youjun Wang | Xiangxi Tujia and Miao Autonomous Prefecture People's Hospital, Jishou, Hunan |
|  | Yingchun Luo | Hunan Provincial Maternal and Child Healthcare Hospital, Changsha, Hunan |
| **Jiangxi** | Xin Zhou | Jiangxi Maternal and Child Health Hospital, Nanchang, Jiangxi |
| **Shanxi** | Liling Shi | Children's Hospital of Shanxi-Women Health Center of Shanxi, Taiyun, Shanxi |
| **Jilin** | Bailing Li | The Hospital for Gynecology and Obstetrics of Changchun City, Changchun, Jilin |
|  | Hongyang Zhang | The First Hospital of Jilin University, Changchun, Jilin |
| **Heilongjiang** | Lichun Zhang | The First Hospital of Qiqihaer, Qiqihaer, Heilongjiang |
|  | Qiuhong Yuan | Mudanjiang Maternal and Child Healthcare Hospital, Mudanjiang, Heilongjiang |
|  | Ming Chen | Harbin Red Cross Central Hospital, Harbin, Heilongjiang |
| **Inner Mongolia** | Guohui Liu | Inner Mongolia Maternal and Child Healthcare Hospital, Huhhot, Inner Mongolia |
| **Sichuan** | Junling Shen | Panzhihua Maternal and Child Healthcare Hospital, Panzhihua, Sichuan |
|  | Jiaxiang Yang | Sichuan Provincial Hospital for Women and Children |
|  | Hong Luo | Second University Hospital Affiliated to Sichuan University, Chengdu, Sichuan |
| **Shaanxi** | Gao Xinru | The Northwest Women's and Children's Hospital, Xian, Shaanxi |
|  | Jun Li | Xijing Hospital, Xian, Shaanxi |
| **Guizhou** | Yao Tang | The University Hospital of Guizhou Medical University, Guiyang, Guizhou |
|  | Yan Liu | Guiyang Maternal and Child Healthcare Hospital, Guiyang, Guizhou |
| **Ningxia** | Wenna Guan | Yinchuan Maternal and Child Healthcare Hospital, Yinchuan, Ningxia |
|  | Xueqin  Ji | General Hospital of Ningxia Medical University, Yinchuan, Ningxia |
| **Gansu** | Yan Che | Lanzhou University Second Hospital, Lanzhou, Gansu |
|  | Yixuan Wang | Gansu Provincial Maternity and Child Care Hospital, Lanzhou, Gansu |
| **Xinjiang** | Yu Zhang | Maternal and Child Healthcare of Xinjiang Uygur Autonomous Region, Wlumuqi, Xinjiang |
|  | Lan Yu | People’s Hospital of Xinjiang Uygur Autonomous Region, Wlumuqi, Xinjiang |
|  | Yinghui Shi | Municipal Maternal and Child Health Hospital, Kuerle, Xinjiang |
| **Hebei** | JIan Gao | Hebei Provincial General Hospital, Shijiachuan, Hebei |
|  | Conxin Sun | Shijiazhuang Obstetrics and Gynecology Hospital, Shijiazhuang, Hebei |
| **Yunnan** | Yanling Huang | Yunnan Red Cross Hospital/The Second People's Hospital of Yunnan Province, Kunming, Yunnan |
|  | Hang Fu | Yuxi Maternal and Child Health Care Hospital, Yuxi, Yunnan |
|  | Yonghong Ma | The First Affiliated Hospital of Kunming Medical University, Kunming, Yunnan |
|  | Wei Miao | The First People's Hospital of Honghe State, Mongzi, Yunnan |
|  | Yu Lei | Lijiang Maternal and Child Health Hospital, Lijiang, Yunnan |
|  | Juyan Li | Dali Municipal Maternal and Child Health Hospital, Dali, Yunnan |
|  | Hongjian Chen | The Third People's Hospital of Yunnan Province, Kunming, Yunnan |
|  | Baosheng Zhu | The First People's Hospital of Yunnan Province,, Kunming, Yunnan |
|  | LiJuan Lu | Kunming Municipal Maternal and Child Health Hospital, Kunming, Yunnan |
| **Qinghai** | Yingfang Wa | Qinghai Provincial Maternal and Child Healthcare Hospital, Xining, Qinghai |
|  | Wenrong Zhou | Qinghai Red Cross Hospital, Xining, Qinghai |
|  | Lina Sun | The Xining First People's Hospital, Xining, Qinghai |
|  | Shengjun Ma | The Xining Second People’s Hospital, Xining, Qinghai |
| **Shanghai** | Yuqing Zhou | Changning Maternity and Infant Health Hospital, Shanghai |
| **Hainan** | Jie Wang | Hainan Provincial Hospital for Maternal and Children’s Health, Haikou, Hainan |
|  | Xiujuan Tian | Sanya Maternity and Child Care Hospital, Sanya, Hainan |
|  | Yongming Hu | Municipal Hospital of the Maternal and Child Health, Haikou, Hainan |
| **Chongqing** | Suzhen Ran | Chongqing Health Center for Women and Children's Health, Chongqing |
|  | Tao Li | Daping Hospital, Chongqing |
| **Tibet** | Ci Ren Yu Zhen | Lhasa Municipal Hospital for Maternal and Children's Health, Lhasa, Tibet |
|  | La MCR | Xizang ART Maternal and Children’s Hospital, Lhasa, Tibet |

***Table S2:* Demographic information for pregnancies**

|  | Number | Percentage |
| --- | --- | --- |
| Maternal age (years old) |  |  |
| < 20 | 259 | 1·43% |
| 20–24 | 4,465 | 24·60% |
| 25–29 | 7,523 | 41·45% |
| 30–34 | 3,821 | 21·05% |
| 35–39 | 1,535 | 8·46% |
| ≥ 40 | 548 | 3·02% |
| History of gravida |  |  |
| 1 | 11,170 | 61·54% |
| 2 | 4,769 | 26·27% |
| 3 | 1,541 | 8·49% |
| > 3 | 671 | 3·70% |
| History of paragravida |  |  |
| 0 | 14,402 | 79·35% |
| 1 | 3,557 | 19·60% |
| > 2 | 192 | 1·06% |
| Gestational weeks at CHD screening |  |  |
| <14W | 42 | 0·23% |
| 14–17·6W | 203 | 1·12% |
| 18–23·6W | 3,226 | 17·77% |
| 24–27·6W | 7,368 | 40·59% |
| 28–-31·6W | 3,272 | 18·03% |
| 32–35·6W | 1,886 | 10·39% |
| ≥ 36W | 987 | 5·44% |
| Unknown | 1,167 | 6·43% |
| Outcomes of pregnancy |  |  |
| Induced labour | 7,543 | 41·56% |
| Delivery | 4,882 | 26·90% |
| Loss follow up | 5,726 | 31·54% |
| Gestational weeks at termination |  |  |
| <14W | 20 | 0·27% |
| 14–17·6W | 120 | 1·59% |
| 18–23·6W | 1,033 | 13·69% |
| 24–27·6W | 2,191 | 29·05% |
| 28–31·6W | 1,184 | 15·70% |
| 32–35·6W | 621 | 8·23% |
| ≥ 36W | 233 | 3·09% |
| Unknown | 2,141 | 28·38% |
| Gestational weeks at delivery |  |  |
| 28–36·6W | 206 | 4·22% |
| 37–41·6W | 1,367 | 28·00% |
| ≥ 42W | 2 | 0·04% |
| Unknown | 3,307 | 67·74% |
| Fetal weight at termination |  |  |
| < 500g | 1,105 | 14·63% |
| 500–999g | 2,369 | 31·37% |
| 1,000–1,499g | 937 | 12·41% |
| 1,500–1,999g | 520 | 6·89% |
| ≥ 2,000g | 477 | 6·32% |
| Unknown | 2,143 | 28·38% |
| Fetal weight at full-term birth |  |  |
| < 1,999g | 17 | 0·35% |
| 2,000–2,499g | 102 | 2·09% |
| 2,500–2,999g | 354 | 7·24% |
| 3,000–3,499 | 612 | 12·52% |
| ≥ 3,500g | 492 | 10·06% |
| Unknown | 3,312 | 67·74% |
| Gestational diabetes |  |  |
| Yes | 242 | 1·33% |
| No | 7,885 | 43·44% |
| Unknown | 10,024 | 55·23% |
| Maternal autoimmune disease |  |  |
| Yes | 77 | 0·42% |
| No | 7,849 | 43·24% |
| Unknown | 10,225 | 56·33% |
| Exposure to teratogen during pregnancy |  |  |
| Yes | 627 | 3·45% |
| No | 1,538 | 8·47% |
| Unknown | 15,986 | 88·07% |
| Family CHD history |  |  |
| Yes | 106 | 0·58% |
| No | 7,129 | 39·28% |
| Unknown | 10,916 | 60·14% |
| Serological screening for Down syndrome |  |  |
| Low risk | 3,098 | 17·07% |
| High risk | 587 | 3·23% |
| Unknown | 14,466 | 79·70% |
| Causes |  |  |
| Teratogen | 627 | 28·96% (627/2,165) |
| Gestational diabetes | 242 | 2·98% (242/8,127) |
| Family CHD history | 106 | 1·47% (106/7,235) |
| Maternal autoimmune disease | 77 | 0·97% (77/7,926) |
| Nuchal translucency (NT) |  |  |
| Normal | 270 | 1·49% |
| Abnormal | 2,607 | 14·35% |
| Unknown | 15,294 | 84·17% |

CHD=congenital heart defect.


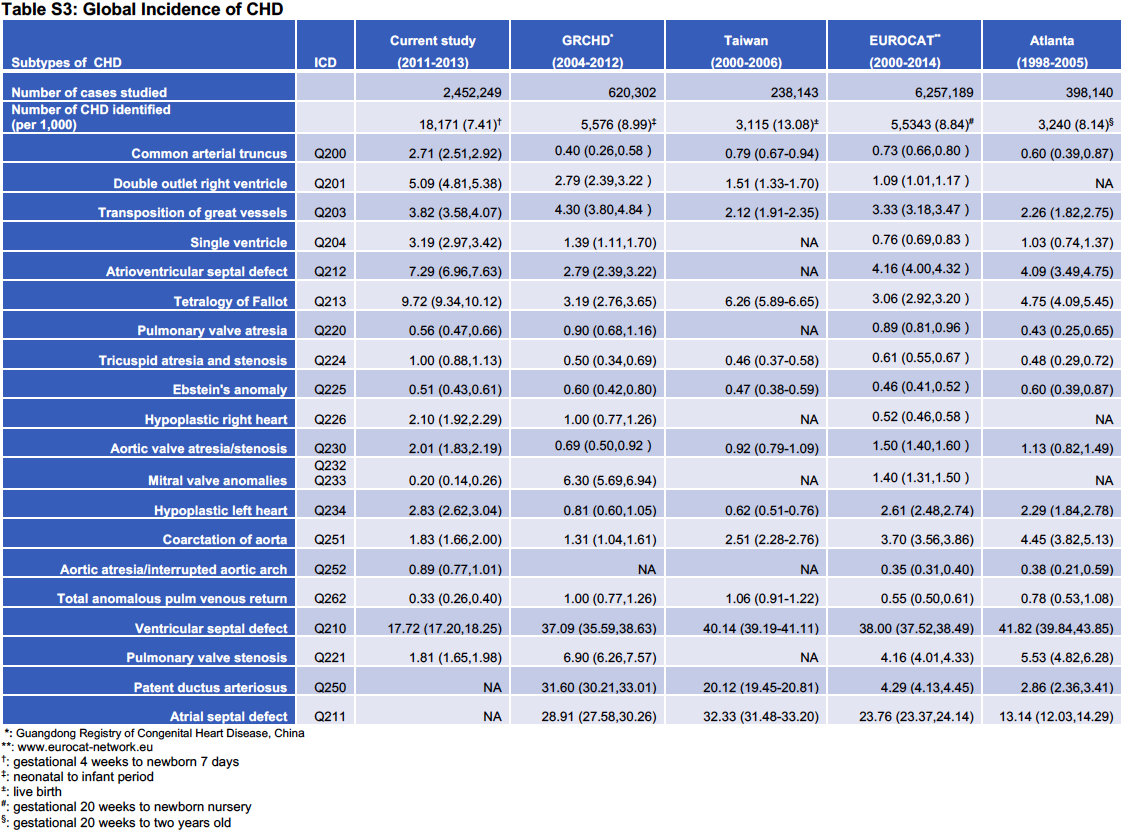


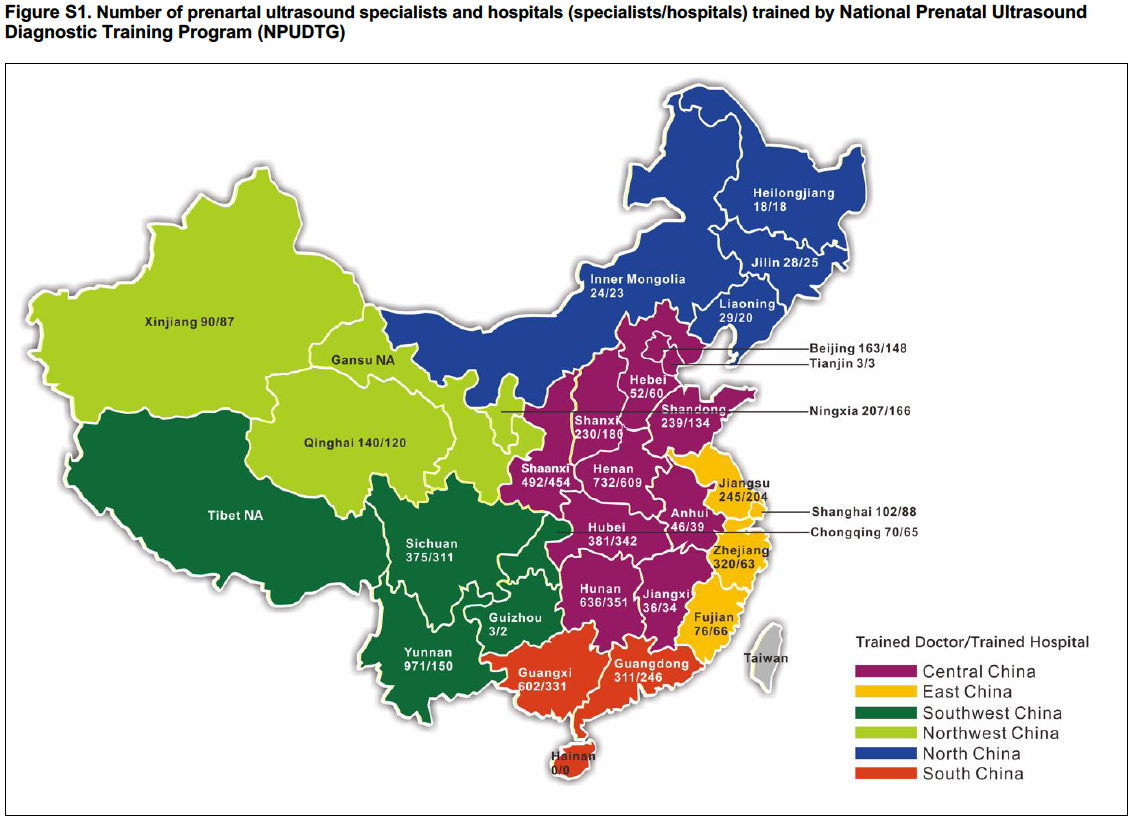


**Figure S1. Prenatal ultrasound professionals trained in the Ministry of Health (MOH)–credentialed National Prenatal Ultrasound Diagnostic Training Centers (NPUDTCs).** Starting in 2006, a national program was developed to offer systemic and intensive training to clinicians who perform prenatal ultrasonography.


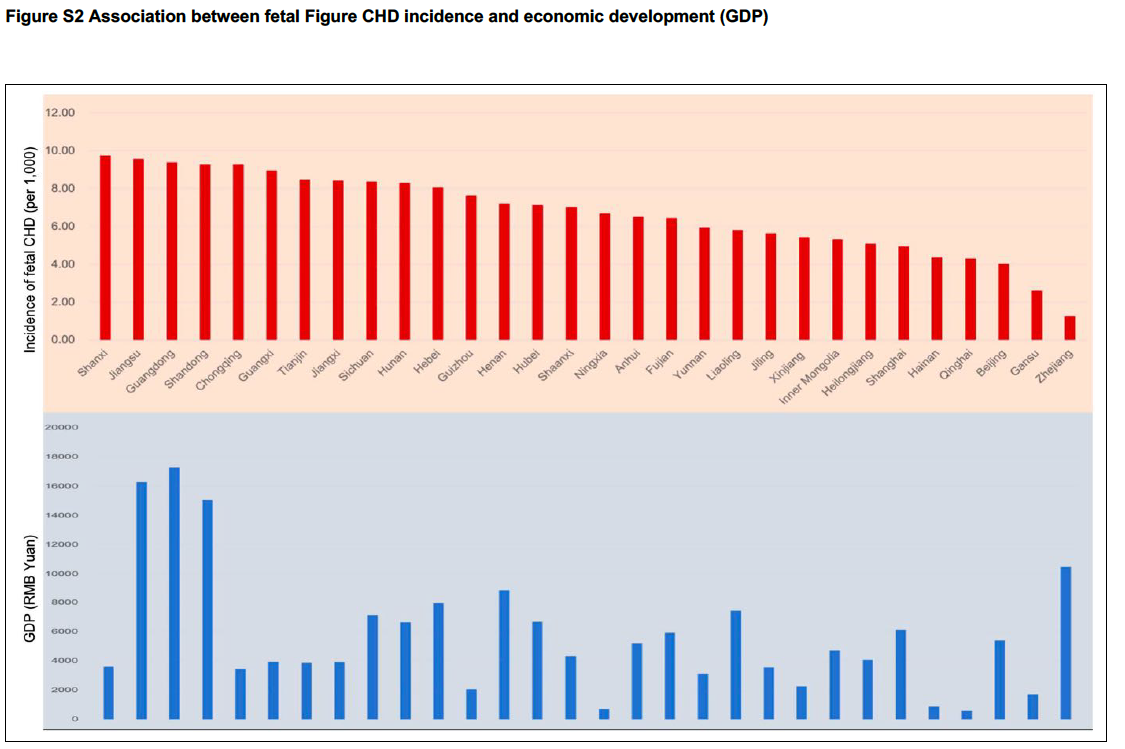


**Figure S2. Association of fetal CHD and economic development.** The high incidence of fetal CHD in three provinces, Jiangsu, Shandong, and Chongqing, which have high gross domestic product (GDP) per capita, would suggest a direct correlation between fetal CHD and GDP per capita. However, in the economically developed Zhejiang province, the incidence of fetal CHD was the lowest in the country. The highest rate of fetal CHD was observed in Shanxi province, which once had the highest rate of neural tube defects in the country and whose GDP per capita is not high. The labels on the X-axis for each province in the lower panel (GDP) are synchronized with the labels in the upper panel (Incidence).
